# Spatiotemporal 7q11.23 protein network analysis implicates the role of DNA repair pathway during human brain development

**DOI:** 10.1038/s41598-021-87632-x

**Published:** 2021-04-15

**Authors:** Liang Chen, Weidi Wang, Wenxiang Cai, Weichen Song, Wei Qian, Guan Ning Lin

**Affiliations:** 1 Shanghai Mental Health Center,’ Shanghai Jiao Tong University School of Medicine, School of Biomedical Engineering, Shanghai Jiao Tong University, Shanghai, 200030 China; 2 Shanghai Key Laboratory of Psychotic Disorders, Shanghai, 200030 China

**Keywords:** Computational biology and bioinformatics, Genetics

## Abstract

Recurrent deletions and duplications of chromosome 7q11.23 copy number variants (CNVs) are associated with several psychiatric disorders. Although phenotypic abnormalities have been observed in patients, causal genes responsible for CNV-associated diagnoses and traits are still poorly understood. Furthermore, the targeted human brain regions, developmental stages, protein networks, and signaling pathways, influenced by this CNV remain unclear. Previous works showed GTF2I involved in Williams-Beuren syndrome, but pathways affected by GTF2I are indistinct. We first constructed dynamic spatiotemporal networks of 7q11.23 genes by combining data from the brain developmental transcriptome with physical interactions of 7q11.23 proteins. Topological changes were observed in protein–protein interaction (PPI) networks throughout different stages of brain development. Early and late fetal periods of development in the cortex, striatum, hippocampus, and amygdale were observed as the vital periods and regions for 7q11.23 CNV proteins. CNV proteins and their partners are significantly enriched in DNA repair pathway. As a driver gene, GTF2I interacted with PRKDC and BRCA1 to involve in DNA repair pathway. The physical interaction between GTF2I with PRKDC was confirmed experimentally by the liquid chromatography-tandem mass spectrometry (LC–MS/MS). We identified that early and late fetal periods are crucial for 7q11.23 genes to affect brain development. Our results implicate that 7q11.23 CNV genes converge on the DNA repair pathway to contribute to the pathogenesis of psychiatric diseases.

## Introduction

Copy number variants (CNVs) are alterations in the number of copies of a genomic DNA region; these alterations can include duplications and deletions^1,2^. CNVs have been shown to act as significant risk factors for complex disorders (e.g., neurodevelopmental and autoimmune) in humans^3^. Previous studies have shown that the duplication or deletion of 7q11.23 has been associated with several psychiatric disorders^4^. For example, the duplication of 7q11.23 can lead to the development of autism spectrum disorder (ASD) or attention deficit hyperactivity disorder^5^, and the deletion of 7q11.23 would cause a specific neurodevelopmental disorder, Williams–Beuren syndrome (WBS). In addition, the microcephaly has been observed recurrently in carriers of the 7q11.23 deletion^6^, while, the macrocephaly clinical phenotype has been observed sometimes in duplication carriers^7^.

The intricacy and highly polygenic characteristics of psychiatric disorders hamper our ability to identify the underlying mechanisms of action with regard to their genetic risk factors. One way of addressing this issue is by analyzing omics data to identify a diverse range of genetic causes. For example, a previous transcriptomic study of the 7q11.23 CNV in patient-derived induced pluripotent stem cell (iPSC) lines suggested alterations in gene expression and related signaling pathways^8^. Furthermore, one gene with a 7q11.23 deletion/duplication, general transcription factor II-I (*GTF2I*), was found to play an important role in the differentiation of iPSCs into disease-relevant lineages^8^. Although a related molecular network was established in this previous study, the network was static which only one developmental period was investigated and the related signal pathways are still not clear.

A different approach to addressing this issue is based on creating animal or cell models to identify the related molecular and cellular mechanisms. For instance, mice with a heterozygous deletion of GTF2I or GTF2IRD1 show defects in skeletal and craniofacial^9^. In addition, the embryos of these mice present with a small head; this is consistent with the clinical phenotype of patients carrying a 7q11.23 deletion. Nevertheless, the signaling pathways affected by these CNV genes remain unknown. Replication factor C subunit 2 (RFC2), another 7q11.23 gene, encodes a subunit of the replication factor C (RFC) complex^10^ and is known to play a role in ATR signaling^11^. Haploinsufficiency for RFC2 led to G2/M checkpoint arrest after DNA damage^12^. However, little is known about how genes with the 7q11.23 deletion/duplication may affect neurodevelopmental disorders because these genes are involved in multiple developmental stages and within different tissues. Hence, genes exhibiting 7q11.23 deletion/duplication play different roles in different developmental stages and different anatomic structures.

CNVs have been reported to modulate gene expression, which, ultimately, might affect disease predisposition or clinical phenotypes^13^. Several studies have investigated CNV pathogenesis in psychiatric disorders by constructing a static topological network based on a single developmental stage^14^. Within different developmental periods, protein expression can change, as can protein–protein interactions (PPIs)^15^. Nevertheless, protein expression is a dynamic process that can occur differently across different anatomical areas^16^. In addition, multiple studies mentioned above focused only on one or two genes and were unable to demonstrate how the 7q11.23 CNV is involved in brain development and psychiatry disorders. Although phenotypic abnormalities have been observed in patients and animal models, the targeted brain regions, periods, protein networks, and signaling pathways, influenced by this CNV remain unclear.

Protein–protein interactions (PPIs) are pivotal for most biological processes. Analysis of PPI networks has become a signifiant method in systems biology. In fact, a protein interaction network frequently refers to physical PPIs. Previous studies reported Robust correlations between higher co-expression and protein interaction^17^. In general, PPIs cannot occur unless proteins are in the same cell components simultaneously. The expression levels of proteins vary according to development stages or conditions, and the PPIs are dynamic as well. For this reason, PPIs could be confirmed by co-expression data. Therefore, integrating gene expression data with PPIs can uncover protein interactions at different developmental periods and in different anatomical areas. Analyses of molecular networks can reveal biological modularity and complex signaling pathways^18^. Previous studies discovered the pathogenesis of CNVs or candidate genes by constructing dynamic protein–protein interaction (PPI) networks according to alterations of protein expression in different anatomical areas and during different developmental periods^19,20^. The specific brain regions, developmental periods, pathways impacted by CNVs or candidate genes can be investigated by dynamic networks^23,24^. To investigate the specific brain regions, developmental periods, signal pathways impacted by 7q11.23 CNV, we constructed a dynamic spatiotemporal network of genes exhibiting the 7q11.23 deletion/duplication by integrating data from the human brain developmental transcriptome with physical interactions of 7q11.23 proteins.

DNA repair have been identified that contribute to the development of numerous neurological disorders^21^. For instance, DNA damage and DNA repair involve in Schizophrenia, Intelligent disability (ID), Autism, Alzheimer's disease and Parkinson’s disease^22,23^. Polymorphisms in DNA repair genes associated with the development of schizophrenia^24^. Previous works showed that impaired DNA repair followed by apoptosis in the developing cortex result in microcephaly^25^. A strong correlation between neurodegenerative diseases and the DNA repair defects was revealed in neurons by previous works^26,27^.

Our study demonstrated that 7q11.23 proteins interact with their partners mainly in three spatiotemporal intervals, and that the interaction patterns change across these intervals. We identified that striatum, hippocampus, and amygdala are crucial regions for the interactions between 7q11.23 proteins and their partners in early and late fetal periods. Our results suggested that the DNA repair pathway is crucial for the 7q11.23 CNV genes to contribute to the pathogenesis of psychiatric diseases. In addition, our results indicated that GTF2I plays a key role in a dynamic network by interacting with DNA-dependent protein kinase catalytic subunit (PRKDC) and Breast cancer type 1 susceptibility protein (BRCA1). We undertook co-immunoprecipitation (Co-IP) experiments and liquid chromatography-tandem mass spectrometry (LC–MS/MS) to identify the interactions between GTF2I and PRKDC.

## Results

### Construction of a spatiotemporal interaction network for 7q11.23

**Figure 1:**
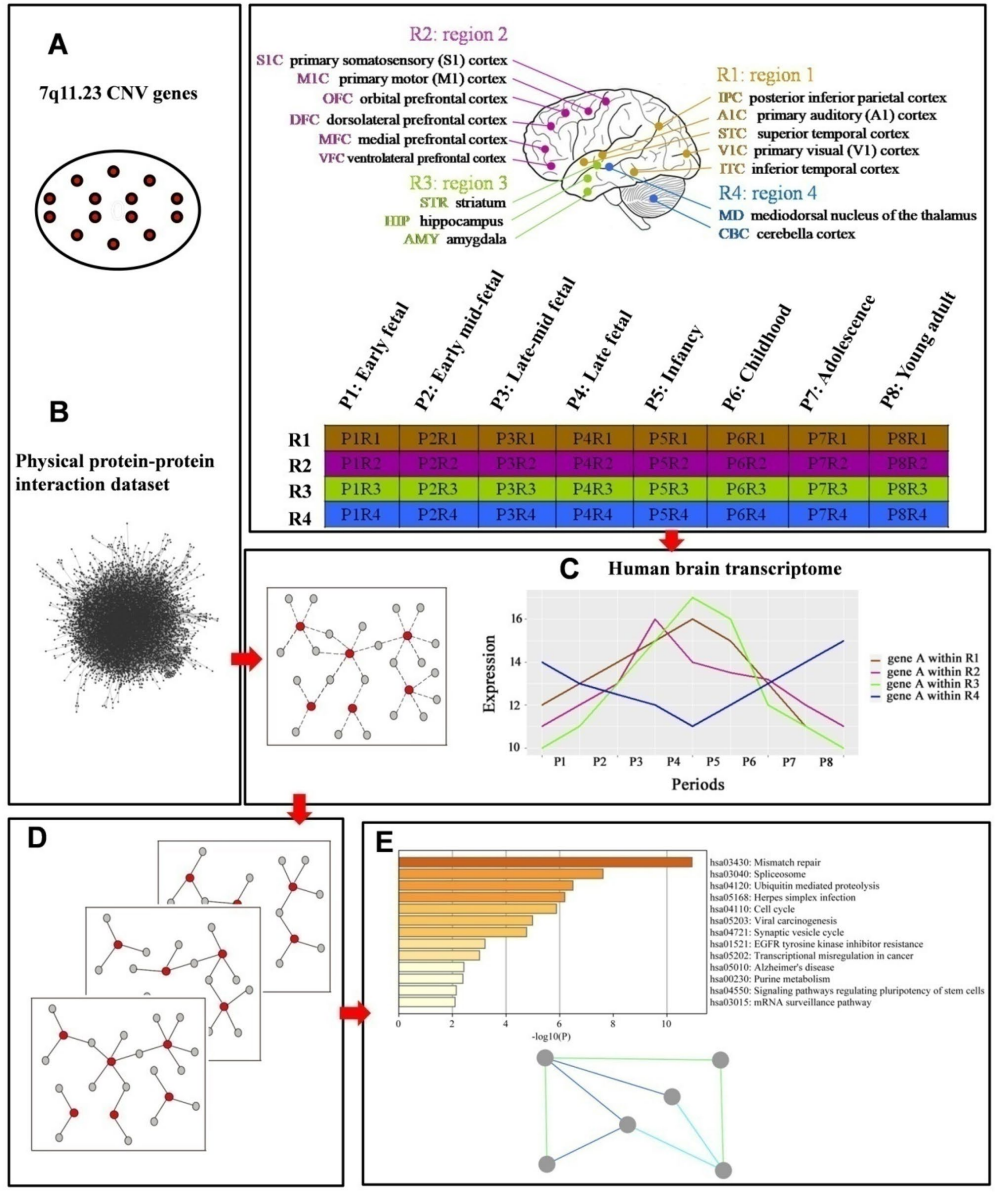
A flow-chart shows the plan of work involved in this research study. (**A**) Twenty-three 7q11.23 CNV genes expressed in the brain were identified. (**B**) Physical protein–protein interaction dataset combined with 7q11.23 CNV genes to construct CNV protein–protein interactions (PPIs). (**C**) 7q11.23 CNV PPIs combined with the Human brain transcriptome dataset. (**D**) 7q11.23 spatiotemporal co-expression PPIs network was established. (**E**) Functional enrichment analysis and functional module analysis were performed.

In general, PPIs take place only if proteins are located in the same cell components simultaneously^28^. A range of studies has demonstrated robust correlations between higher co-expression and protein interaction across most cellular conditions^17^. Thus, the combination of data relating to gene expression and protein interaction could reveal protein interactions at different developmental stages and in different anatomical regions. To investigate the regulatory roles of the 7q11.23 CNV in the development of the human brain, we extracted 21 brain-expressed genes located on the chromosomal region of 7q11.23 encompassing ~ 1.4 Mb (chromosome 7: 72.4–73.4 Mb) (Supplementary Table S1 and constructed dynamic networks by integrating spatiotemporal gene co-expressions of the developing human brain with the physical PPIs of 7q11.23 proteins (Fig. 1).

A protein interaction network frequently refers to physical PPIs. To construct human brain spatiotemporal network of 7q11.23 CNV proteins, we download the human brain transcriptome data and human physical protein–protein interaction data. We obtained gene expression data in the brain from BrainSpan (www.brainspan.org) and partitioned different regions and periods (Methods), as described by Lin and colleagues^20^ (Supplementary Table S2,S3). We defined 32 spatiotemporal intervals based on eight periods of brain development (P1–P8) and four brain regions (R1–R4) and excluded P3R4 (P3, late mid-fetal; R4, mediodorsal nucleus of the thalamus and cerebella cortex) due to insufficient data for analyses. Three control datasets were used for controlling biases in the analyses: (i) physically interacting partners interacting with proteins of common CNVs identified in the 1000 Genomes Project; (ii) all possible pairs between 7q11.23 CNV genes and brain expressed genes; and (iii) all brain-expressed proteins interacting with their physically interacting partners.

### 7q11.23 co-expressed interacting protein pairs are enriched in early and late fetal periods

**Figure 2:**
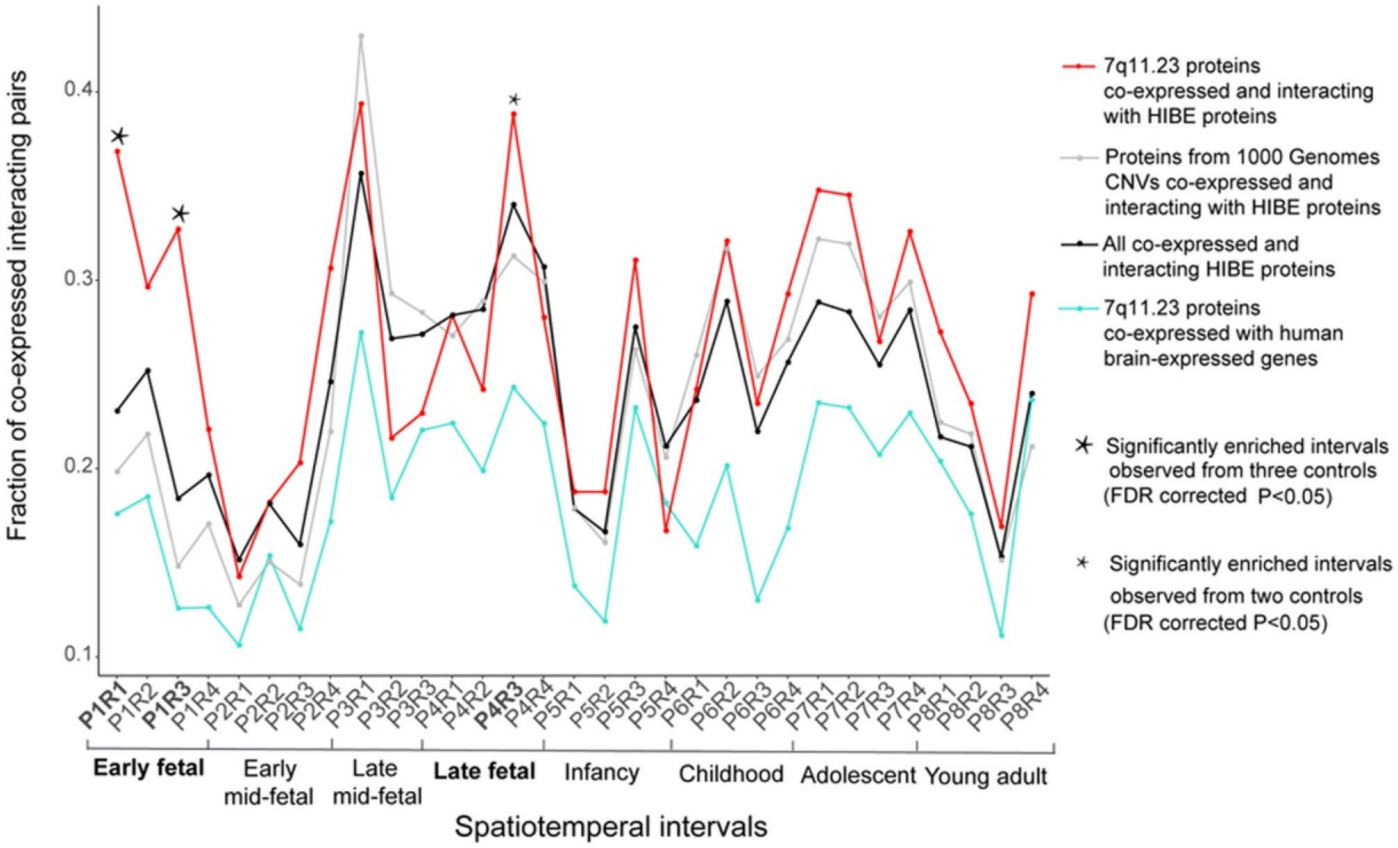
The 7q11.23 co-expressed interacting protein pairs are significantly enriched in three spatiotemporal intervals. The fractions of protein pairs from 7q11.23 CNV co-expressed and interacting with HIBE proteins (red line), all co-expressed and interacting HIBE proteins (black line), proteins from 1000 Genome Project CNVs co-expressed and interacting with HIBE proteins (dark gray line), and 7q11.23 CNV proteins co-expressed with all brain-expressed human genes (aquamarine line). Thirty-one spatiotemporal intervals of brain development are shown on the x-axis. 7q11.23 co-expressed interacting protein pairs are significantly enriched in spatiotemporal intervals (indicated by star symbol) compared with control networks. The statistical enrichment was calculated using Fisher’s exact test, and *P* values were FDR-corrected for multiple comparisons.

To identify significant enrichment of connectivity for 7q11.23 CNV, we calculated fractions of co-expression interacting pairs for 7q11.23 proteins and three control datasets across 31 spatiotemporal intervals. After false-discovery rate (FDR) correction for multiple testing, we observed significantly more co-expressed interacting pairs in two spatiotemporal network intervals than in all three controls: P1R1 (P1: early fetal; R1: parietal, temporal, and occipital cortex; Fisher’s exact test; *p* = 1.05 × 10^−6^, *p* = 3.41 × 10^−15^, *p* = 4.82 × 10^−6^) and P1R3 (P1: early fetal; R3: amygdala, hippocampus, and striatum; Fisher’s exact test; *p* = 1.41 × 10^−8^, *p* = 3.41 × 10^−15^, *p* = 2.19 × 10^−15^ (Fig. 2). Another spatiotemporal interval, P4R3 (P4: late fetal; R3: amygdala, hippocampus, and striatum), showed significantly more co-expressing interacting pairs in 7q11.23 CNV network than two of control networks: (i) co-expressing physical PPIs of common CNVs (*p* = 4.34 × 10^−2^) and (ii) possible pairs between proteins with the 7q11.23 CNV and all brain-expressed proteins (*p* = 2.24 × 10^−7^) (Fig. 2).

### Similarities and differences between the 7q11.23 networks

**Figure 3:**
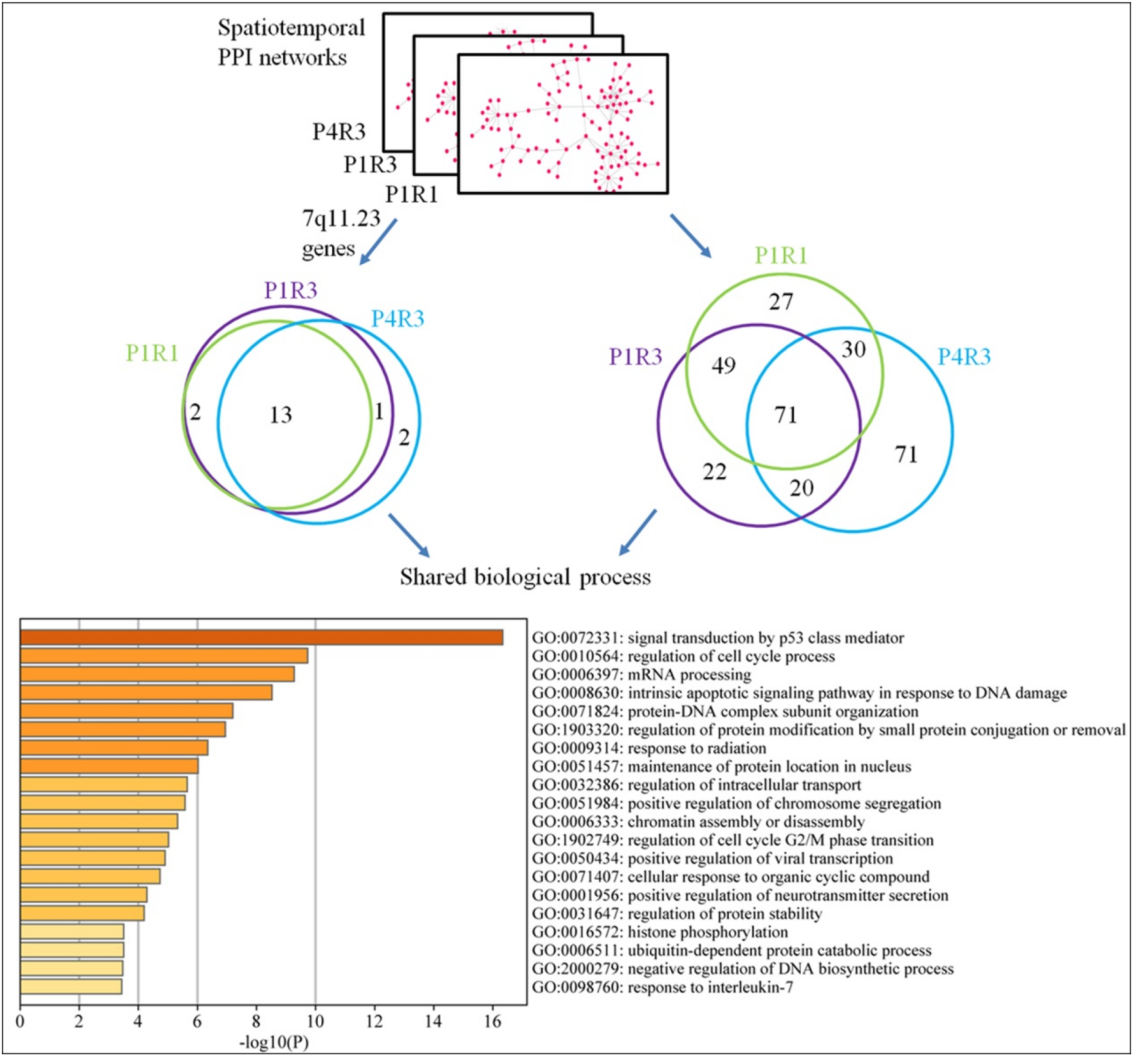
Functional convergence of the 7q11.23 spatiotemporal networks. The overlap of 7q11.23 genes (left Venn diagram) and their co-expressed interacting partners (right Venn diagram) are across three significant spatiotemporal intervals. Top 20 significant enriched biological process GO terms of shared proteins are showed.

Next, we investigated similarities between the three significant networks, by identifying their convergence by computing the fraction of shared proteins in these networks. We observed that 13 of 23 (56%) 7q11.23-CNV proteins and 71 of 290 (24.5%) of their co-expressed interacting partners were shared by all three networks in three intervals: P1R1 (P1: early fetal; R1: parietal, temporal, and occipital cortex), P1R3 (P1: early fetal; R3: amygdala, hippocampus, and striatum) and P4R3 (P4: late fetal; R3: amygdala, hippocampus, and striatum) (Fig. 3). Next, we undertook analyses of functional enrichments on shared CNV genes and shared interacting partners using Metascape (http://metascape.org)^29^. The top-three significant categories for the biological process were “signal transduction by p53 class mediator”, “regulation of cell cycle process”, and “mRNA processing” (Fig. 3).

**Figure 4:**
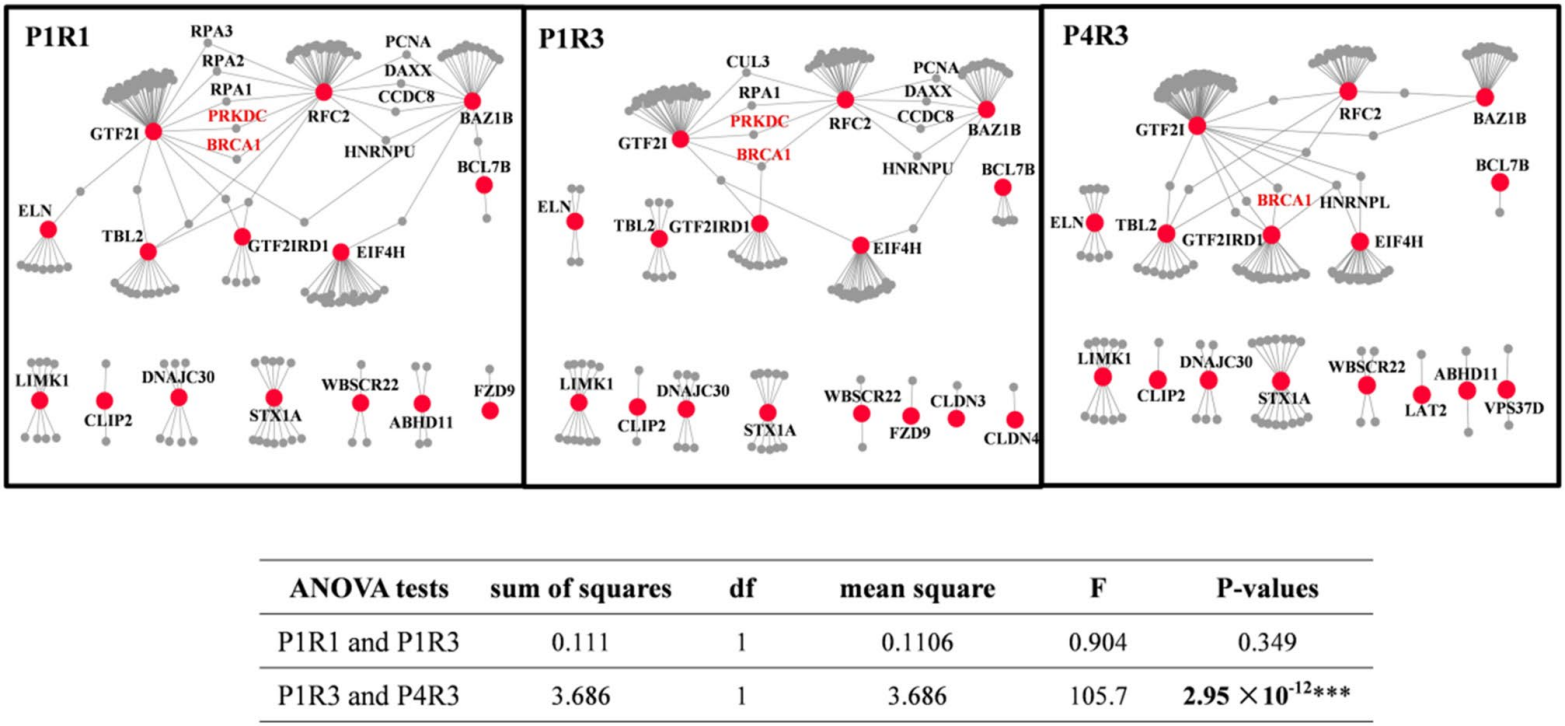
Difference between the 7q11.23 spatiotemporal networks. Spatiotemporal networks were compared across different brain regions within the same developmental period (P1R1 and P1R3) and cross different development periods within the same brain region (P1R3 and P4R3). 7q11.23 genes are shown as red nodes, their co-expressed interacting partners as gray node, and the PPIs between co-expressed genes at a particular developmental period are shown as gray edges. The nodes that lost all edges were removed from the corresponding networks. Significant differences are observed across developmental periods but not across brain regions (ANOVA statistics shown below the graphs).

Next, we compared connectivity of co-expressed interacting protein pairs within the same developmental period (early fetal, P1) or within the same brain region (R3) to identify both topological and functional differences between the spatiotemporal 7q11.23 networks. As noted, we have identified three spatiotemporal networks with significantly enriched co-expressed PPI pairs across different brain regions (R1 and R3) within the same developmental period (early fetal, P1) and also across different developmental periods (early fetal P1 and late fetal P4) within the same region (R3). Network changes were assessed by calculating the fractions of co-expressed PPI pairs unique to one network against the fractions of co-expressed PPI pairs shared among networks (Fig. 4). We noted a significant difference between identical region within different developmental periods (P1R3 and P4R3; ANOVA; *p* = 2.95 × 10^−16^) (Supplementary Table S4). In contrast, no significant difference was observed between the same period within different regions (P1R1 and P1R3, ANOVA; *p* = 0.349) (Supplementary Table S5). All dynamic co-expression PPI networks of 7q11.23 were shown in supplementary Figure S2.

### 7q11.23 networks involved in the regulation of DNA repair and DNA replication

**Figure 5:**
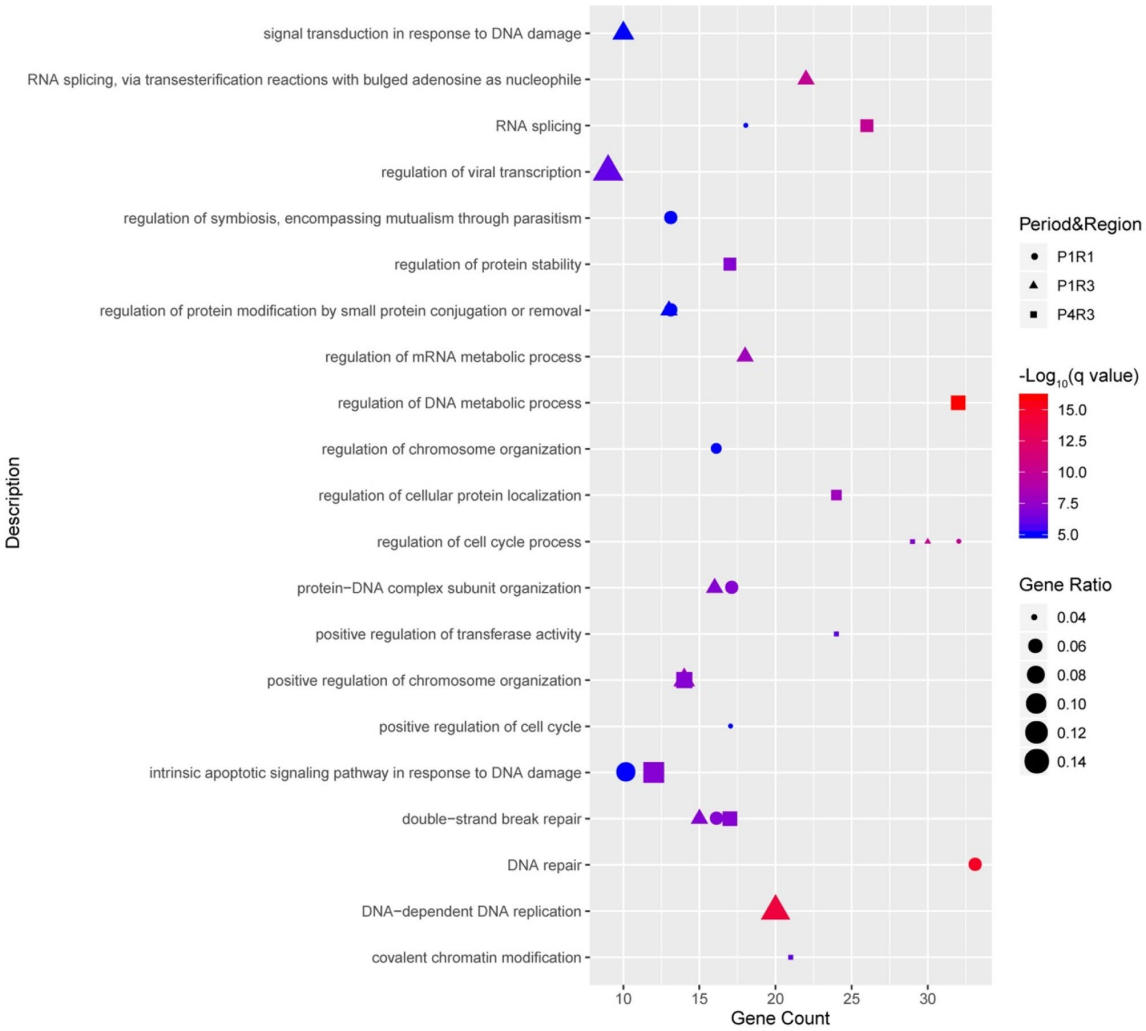
Functional analyses of proteins within three significant intervals, P1R1, P1R3 and P4R3. Dot plot shows top 10 enriched GO terms of biological process for CNV proteins and their partners within three significant intervals.

Next, we set to investigate the biological functions of 7q11.23 proteins and their partners within three dynamic 7q11.23 networks, P1R1, P1R3 and P4R3. Hence, we analyzed functional enrichment of the pathways of related genes using the Gene Ontology (GO) and Kyoto Encyclopedia of Genes and Genomes (KEGG). For 7q11.23 proteins and their partners from the P1R1 network, the top-three significant terms for the biological process were “DNA repair”, “regulation of cell cycle process”, and “double-strand break repair” (Fig. 5). The top-three terms for the biological processes involving 7q11.23 proteins and partners from the P1R3 network were “DNA-dependent DNA replication”, “RNA splicing via transesterification reactions with bulged adenosine as nucleophile”, and “regulation of cell cycle process” (Fig. 5). For 7q11.23 proteins and their partners from the P4R3 network, the top-three significant terms were “regulation of DNA metabolic process”, “RNA splicing”, and “regulation of cellular protein localization” (Fig. 5). Next, we observed that 71 co-expressed and interacting partners of CNV proteins were exclusively from the P4R3 network, not from P1R1 and P1R3 networks, and associated with “signaling pathways regulating pluripotency of stem cells (PSCs)”, “EGFR tyrosine kinase inhibitor resistance”, “cell cycle”, and the “hippo signaling pathway”. (Supplementary Fig. S1). Biological functions of 7q11.23 proteins and their partners within all dynamic networks were shown in supplementary Figure S3.

### Spatiotemporal networks identified driver gene and the DNA repair pathway

Of the three significant networks that we identified above, GTF2I possesses the highest radiality value, indicating that GTF2I is a driver gene and adopts a central position within these networks (Supplementary Table S6). It has been reported that *GTF2I* heterozygotes exhibit microcephaly and retarded growth^9^. Recent studies have also demonstrated that *GTF2I* is involved in neurodevelopment^30^. Notably, the phenotype observed in mice mirrors that observed in humans^31^. As a driver gene within networks, *GTF2I* is a crucial contributor to neuropsychiatric disorders^32,33^. Hence, we investigated the interaction pattern of GTF2I across three significant spatiotemporal networks.

**Figure 6:**
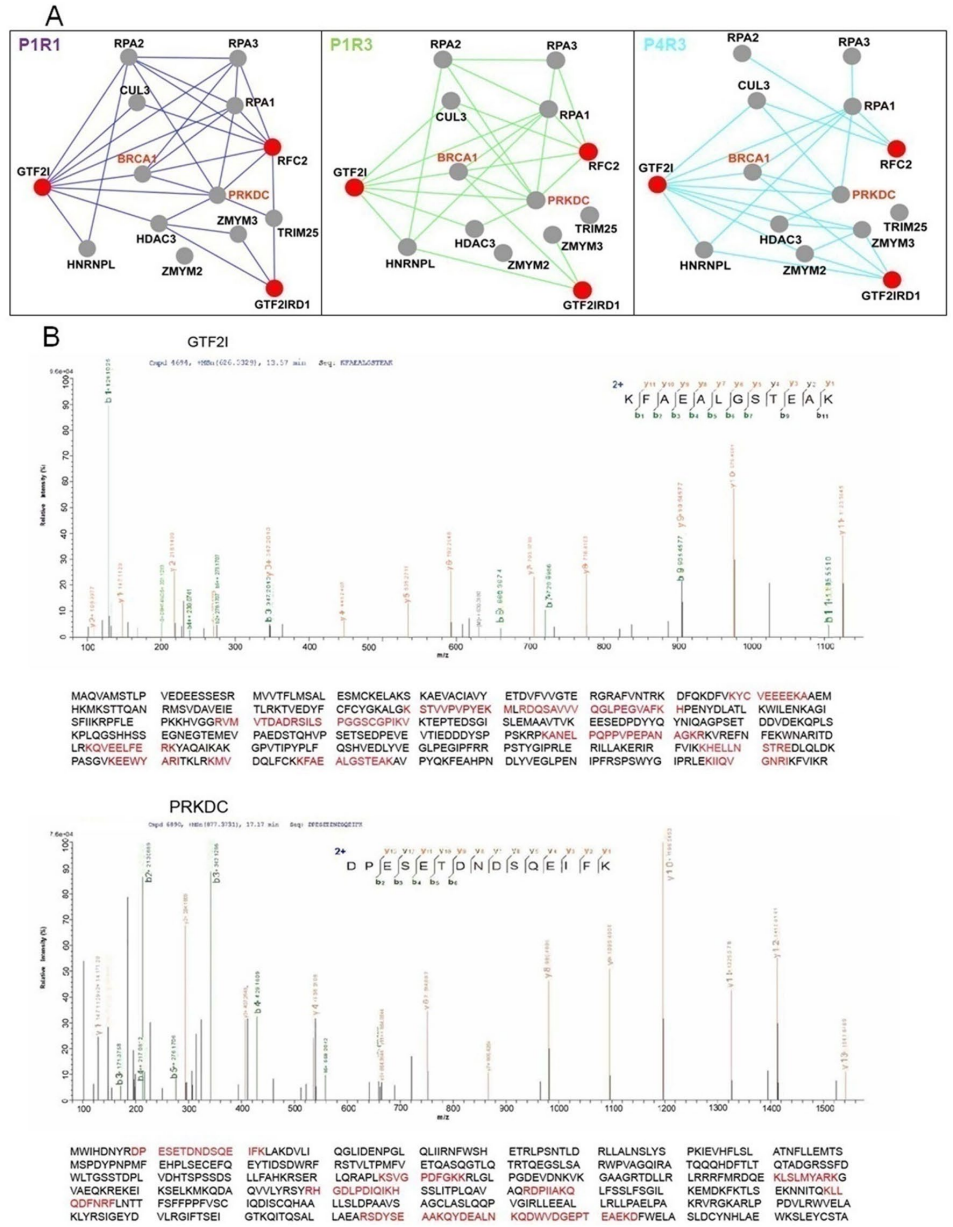
Spatiotemporal networks implicate GTF2I-PRKDC-DDR pathway and proteomic investigation of interaction between GTF2I and PRKDC. (**A**) Dynamic spatiotemporal networks of the GTF2I, GTF2IRD1, and RFC2 interacted with their hub partners. 7q11.23 genes are shown as red nodes, their co-expressed hub partners as gray node, and the PPIs between co-expressed genes at a particular developmental period are shown as gray edges. The nodes that lost all edges were removed from the corresponding networks, and the PPIs between co-expressed genes at a particular developmental period are showed as colored edges (P1R1, blueviolet; P1R3, green; P4R3, turquoises). (**B**) GTF2I and PRKDC were identified and the amino acids highlighted are peptides identified by immunoprecipitation (IP) and LC–MS/MS.

Seventy-three proteins interacted physically and were co-expressed with GTF2I across three spatiotemporal intervals (Fig. 4). Several of these partners were hub proteins that interacted with several CNV proteins. These hub proteins also interacted physically and were co-expressed with each other, thereby forming one functional module (Fig. 6A). These hub partners were PRKDC, BRCA1, ZMYM2, ZMYM3, HDAC3, RPA1, RPA2, and RPA3. Of these partners, PRKDC possesses the highest radiality value in the functional module (Supplementary Table S7). GTF2I interacts with PRKDC, which acts as a “sensor” for double-strand DNA breaks^34,35^. PRKDC lies within the 8q11.21 locus and promotes DNA repair via nonhomologous end-joining (NHEJ)^36^.

GTF2I also interacts with BRCA1, a nuclear phosphoprotein required to repair of double-strand DNA breaks and homologous recombination^37^. Tanikawa M and colleagues showed that GTF2I proteins bind directly to BRCT (the carboxyl-terminal domain of BRCA1)^38^, thus indicating that GTF2I plays an important role in DNA repair. ZMYM2 acts as a transcription factor and is involved in DNA damage response (DDR)^39^. ZMYM3 is a component of histone deacetylase (HDAC)-containing multiprotein complexes. ZMYM3 and HDAC3 involve in a DNA-damage pathway and facilitates DNA repair^40,41^. Furthermore, RPA1, RPA2, and RPA3, are subunits of the heterotrimeric replication protein A (RPA) complex, which is involved in DNA repair and DNA replication.

BRCA1 and PRKDC exhibited higher levels of connectivity in the early-fetal period than in the late-fetal period, thus suggesting that these two hub proteins play a more important role during the early-fetal period. BRCA1 and PRKDC interact with two CNV proteins (GTF2I and RFC2) in P1R1 network. PRKDC interacts with GTF2I and RFC2 within P1R3 network. BRCA1 interacts with three CNV proteins (GTF2I, RFC2 and GTF2IRD1) in P1R3 network. PRKDC interacts with GTF2I in P4R3 network. BRCA1 interacts with GTF2I and GTF2IRD in P4R3 network. Within P4R3 network, the interactions between PRKDC and BRCA1 with RFC2 were not observed. PRKDC and BRCA1 exhibit a similar interaction pattern during early-fetal and late-fetal periods. The interactions between GTF2I and RFC2 with RPA1 exhibit no change during early-fetal and late-fetal stages.

### Validation of the interaction between GTF2I and PRKDC by immunoprecipitation and LC–MS/MS

Next, we investigated the proteins that interact with GTF2I. Hence, the immunocomplex arising from the immunoprecipitation of the control immunoglobulin G (IgG) antibody or Myc antibody was separated by sodium dodecyl sulfate–polyacrylamide gel electrophoresis (SDS-PAGE), followed by staining with Coomassie brilliant blue (CBB). Several unique protein bands were observed consistently in complexes pulled-down by the Myc antibody, but not in the control IgG (supplementary Fig. S2). The interaction between GTF2I and PRKDC was detected by liquid chromatography-tandem mass spectrometry (LC–MS/MS) (Fig. 6B).

### De novo mutations are significantly enriched in spatiotemporal network

De novo mutations have been implicated in various psychiatric disorders as potential disease risks^42^. Thus, we compared all of the interacting partners and 7q11.23 proteins with the de novo mutations observed in psychiatric diseases (supplementary table S8). Proteins from the spatiotemporal 7q11.23 network were enriched significantly in ASD genes (FDR-corrected *p* = 7.21 × 10^−4^). These proteins were also significantly enriched in genes associated with developmental delay (FDR-corrected *p* = 0.0045) and the target genes for fragile X mental retardation protein (FMRP) (FDR-corrected *p* = 0.0017). There was no significant difference between the entire 7q11.23 network and genes for neurodegenerative disease (FDR-corrected *p* = 0.03749) (Supplementary Table S8).

## Discussion

We constructed a dynamic spatiotemporal network for the 7q11.23 CNV, a crucial risk factor for psychiatric disorders. Importantly, the spatiotemporal network indicated that 7q11.23 CNV genes played a crucial role in three intervals: P1R1 (early fetal, frontal lobe), P1R3 (early fetal, striatum, hippocampus, and amygdale) and P4R3 (late fetal, striatum, hippocampus and amygdale). The early-fetal and late-fetal periods were the vital periods for 7q11.23 CNV proteins to affect human brain development. These results agree with studies showing that *GTF2I* haplo-insufficient mice exhibit small brain and neural defects during embryonic development^9,43^.

Sanders SJ and colleagues showed that the 7q11.23 CNV is involved in the pathogenesis of ASD^19^. We observed that mutations in the proteins from the spatiotemporal 7q11.23 network were significantly enriched in ASD genes and the genes associated with developmental delay. Our study suggests that the hippocampus, amygdale, striatum, and frontal lobe, are crucial regions affected by CNV genes. This result is in accordance with a previous report that showed the amygdala, cortex, and hippocampus to be abnormal in mice exhibiting a heterozygous deletion of 7q11.23 critical regions^44^. These results indicate the 7q11.23 CNV plays a significant role in developing the amygdala, cortex, and hippocampus of the human brain.

Our data also suggest that *GTF2I* is a candidate driver gene within the significant networks. Deurloo MHS and colleagues previously showed that *GTF2I* plays a pathological role in WBS^30^. Microcephaly and retarded growth were observed in mice that were heterozygous for *GTF2I*^9^. Importantly, one of the pathways our study suggests as being most likely impacted by the 7q11.23 CNV is the DNA repair pathway. We observed that GTF2I and RFC2 interacted with PRKDC, a vital hub partner with the highest radiality value. Through proteomic analyses, we identified that GTF2I interacted with PRKDC. As a significant hub partner, PRKDC encodes the catalytic subunit of DNA-dependent protein kinase and is associated with repairing DNA double-strand breaks by NHEJ^45^. Mice exhibiting the homozygous deletion of PRKDC show increased levels of apoptosis in the neocortex^46^. PRKDC maintains the integrity of the genome and plays a neuroprotective role in the nervous system following DNA damage^46^. Dysregulation of the DNA repair pathway has been shown to be a pivotal cause of neurodevelopmental disease. A common pathogenic mechanism of microcephalic disorders is defective DNA repair^47^. O'Driscoll M and co-workers previously showed that PRKDC mutations could lead to microcephaly^48^. Our present data further implicate that GTF2I interacts with PRKDC to involve in a pathway of DNA-damage repair. Based on the assessment of spatiotemporal networks, the interaction between GTF2I and PRKDC was observed within the frontal lobe (R1) and striatum, hippocampus, amygdale (R3) during early-fetal (P1) and late-fetal (P4) periods.

Our analyses observed that several hub partners from dynamic networks are involved in the DNA repair pathway. DNA repair pathways play a pivotal role in the maintenance of genomic integrity^49^. DNA repair is critical during the early stage of proliferation as progenitor cells expand and differentiate to generate the nervous system^50^. Previous studies uncovered that untimely repair of DNA damage before the onset of mitosis might result in a cell cycle arrest^51^. Lack of repair of double-stand breaks usually leads to apoptosis, and the consequent loss of neurons by apoptosis could result in neurodegenerative disorders^52^. Apoptosis during neurogenesis is a significant mechanism of microcephaly although other mechanisms still a possibility^53^. Previous works reveal that DNA repair defects might cause neurodegeneration by impairing the transcription of critical neural genes^54^. Our results suggest that BRCA1 interacts with three CNV proteins, GTF2I, GTF2IRD1, and RFC2. Previous studies proved that GTF2I interacts with BRCA1 in vivo and improves the transcriptional activation of BRCT^38^. The BRCA1-associated genome surveillance complex is related to the recognition and repair of DNA damage. BRCA1 affects the embryonic development of mouse brains and postnatal mouse brain size^55^. The deletion of BRCA1 in neural progenitors leads to the disruption of normal differentiation. Pyramidal neurons originating from BRCA1-knockout mice lack the typical radial orientation of apical dendrites^55^. GTF2I and RFC2 interact with RPA, which consists of three subunits: RPA1, RPA2, and RPA3. RPA activates ATR-mediated pathways and is involved in ATR-dependent DDR and cell-cycle arrest. O'Driscoll M and colleagues suggested a causal relationship between dysfunction in ATR signaling and developmental delay. GTF2I and GTF2IRD1 heterozygotes exhibit microcephaly and neural defects^9,43^. GTF2I and GTF2IRD1 can interact with HDAC3, ZMYM2, and ZMYM3. HDAC3 mediates the deacetylation of histones and plays an important role in cell survival and cell-cycle progression. ZMYM2 and ZMYM3 are members of the MYB transcription factor family. ZMYM2 involves in DDR pathway and transition of the G1/S phase of the cell cycle. ZMYM3 facilitates DNA repair by regulating BRCA1 localization at damaged chromatin^40^. Our results suggest that GTF2I interacts with these hub proteins and involves in the DNA-repair pathway to affect brain development.

## Conclusions

We identified that striatum, hippocampus, and amygdala are crucial regions for establishing connectivity between 7q11.23 proteins and their partners in early and late fetal periods. Our results suggested that the DNA repair pathway is crucial for the 7q11.23 CNV genes to contribute to the pathogenesis of psychiatric diseases. GTF2I interacted with BRCA1, PRKDC, and other partners to involve in the DNA repair pathway, and demonstrated its important role in brain development.

## Methods

### Identification of 7q11.23 genes, collection of the human brain transcriptome data and PPI data

Twenty-three genes are located on the regions encompassing ~ 1.4 Mb (chromosome 7: 72.4–73.4 Mb)^5,56^. *FKBP6* and *WBCRS28*, were excluded because these two genes are not expressed (log_2_-intensity < 0.4) in the human brain (supplementary table 1). A protein interaction network generally refers to physical PPIs. To construct human brain dynamic networks of 7q1.23 CNV, the human brain transcriptome data and human physical PPI data were downloaded. Our study utilized region- and time-specific transcriptomic data from the developing human brain; these data were acquired from BrainSpan (www.brainspan.org/, RNA-Seq Gencode v3c summarized to genes). The normalized reads per kilobase per million (RPKM) expression data from 578 developing brain samples derived from 16 cortical and subcortical structures across 13 developmental stages. To reduce noise, we removed genes with a log_2_-intensity < 0.4 in all samples and with a coefficient of variation < 0.07. Consequently, 15,095 genes were regarded to be expressed in the brain. Protein–protein interaction data were downloaded from Biogrid (https://downloads.thebiogrid.org/BioGRID/Release-Archive/BIOGRID-3.4.161/). Biogrid 3.4.161 was downloaded in May 2018 (BIOGRID-ORGANISM-3.4.161.tab2). The human PPIs were used (BIOGRID-ORGANISM-Homo_sapiens-3.4.161.tab2.txt). Only physical PPIs were reserved. Following the removal of redundant and self-interacting data, 241,123 pairs were retained. Physical PPIs were combined with the human brain transcriptome to construct a brain-expressed human interactome (HI_BE_).

### Construction of the spatiotemporal protein network

Human-brain transcription data were divided by 13 dissection stages from 16 anatomic structures^16^. We defined eight non-overlapping periods by merging the developmental stages ranging from 8 post-conception weeks to 39 years-of-age, and by eliminating samples from those that were 40 years-of-age due to their limited size (Supplementary Table S2). According to anatomical and functional similarities, anatomical structures were divided into four areas (Supplementary Table S3). Therefore, 31 spatiotemporal protein networks were constructed following the removal of one region from P3 (P3R4) due to a lack of RNA-sequencing data. Genes within the 7q11.23 CNV were mapped to the HI_BE_ network to establish a static network. Spatiotemporal expression data were incorporated with static PPI networks and the Spearman correlation coefficient was calculated. Interactions were corroborated only if the Spearman correlation coefficient was > 0.5. Consequently, 31 networks were constructed. Cytoscape software (version 3.7.2; http://www.cytoscape.org/) was used to visualize the network or specific module, and to calculate topological parameters. Fisher’s exact test is used to determine whether co-expressing interacting pairs are significantly more in 7q11.23 CNV networks than control networks.

### Enrichment analyses in three spatiotemporal networks

One-way analysis of variance (ANOVA) was performed to assess differences between 7q11.23 networks from the same developmental period (P1R1 and P1R3) or from the same anatomical area (P1R3 and P4R3). Topological features were defined for each gene with the 7q11.23 CNV: the ratio of interacting partners unique to one network and the ratio of interacting partners shared by two networks. Significant differences were identified by ANOVA. Genes within specific networks were analyzed by online tools in Metascape^29^. Functional enrichment was investigated in three GO categories: biological process, molecular function, and cellular component. Terms with *p* < 0.01, a minimum count of 3, and an enrichment factor > 1.5 (the enrichment factor was defined as the ratio of the observed count to the count expected by chance) were collected and grouped into clusters based on their membership similarities. More specifically, P-values were calculated based on the cumulative hypergeometric distribution. The Q-value was calculated using the Benjamini–Hochberg correction to account for multiple testing. The ASD risk gene set includes 239 genes. This gene set was obtained from a previous report^57^. FMRP target genes were extracted from a previous publication (839 genes)^58^. Voltage-gated calcium channel complexes proteins were from a previous study by Catrin Swantje Müller (206 genes)^59^. Developmental delay genes were obtained from a previous report (1291 genes)^60^. Abnormal nervous system electrophysiology (MP: 0,002,272) and abnormal long-term potentiation (MP: 0,002,207) were download from the Mouse Genome Informatics (MGI) database (http://www.informatics.jax.org) ^59^. Differences between the interacting proteins from 7q11.23 spatiotemporal networks and 20,240 genes were analyzed by Fisher’s exact test. P-values were corrected using the Benjamini–Hochberg method.

### Cell culture and transfection

HEK293T cells were cultured in Dulbecco’s modified Eagle’s supplemented with 10% fetal bovine serum, 1% penicillin–streptomycin, and maintained in a humidified incubator at 37 °C in an atmosphere containing 5% CO_2_. For cell transfection, 1.5 × 10^6^ cells were seeded into a 10-cm dish until they reached 80–90% confluency. Transfections were undertaken using the jetPRIME Transfection Reagent with pCMV6-entry-myc-GTF2I. After 48 h, cells were washed with phosphate-buffered saline, collected, and resuspended in lysis buffer (20 mM Tris–Cl, 5 mM EDTA, pH 7.4, 150 mM NaCl, 1% Triton X-100, and 10% (*v/v*) glycerol), supplemented with phenylmethylsulfonyl fluoride (1 mM) and complete protease inhibitor cocktail. Proteins in the supernatant were collected by centrifugation at 13,000 rpm for 15 min at 4 °C; 5% of the supernatant was saved so that it could act as an input control. The remaining cell lysates were immunoprecipitated with anti-Myc antibody (M4439, Sigma–Aldrich, Saint Louis, MO, USA) or normal mouse IgG (I5381, Sigma–Aldrich) rotated for 12 h at 4 °C. Subsequently, cell lysates were added to 40 μL of protein G beads and rotated overnight at 4 °C. Immunocomplexes were washed three times in lysis buffer and boiled with 5 × SDS sample buffer; the supernatant was then collected by centrifugation at 12,000 rpm for 1 min at 4 °C. Supernatants were resolved by 4–20% polyacrylamide Tris–glycine SDS gels and stained by Coomassie Brilliant Blue. Protein bands were excised (120 kDa and above 270 kDa).

### Peptide preparation and LC–MS/MS

First, gels were de-stained with 50% (*v/v*) methanol and vortexed vigorously for 30 min. After de-staining, gel pieces were washed in water for 15 min. Gel pieces were then dehydrated in 100% acetonitrile for 10 min and dried in a vacuum centrifuge. The disulfide bonds of proteins were then reduced with dithiothreitol (10 mM) and alkylated with iodoacetamide (55 mM). Next, gel pieces were washed with 50% (*v/v*) acetonitrile, NH_4_HCO_3_ (25 mM) and dehydrated with 100% acetonitrile. Gel pieces were digested with trypsin in NH_4_HCO_3_ (25 mM). Peptides were extracted with 50% (*v/v*) acetonitrile and 1% (*v/v*) trifluoroacetic acid. Free peptides were dried using a vacuum centrifuge and separated using a liquid chromatograph (Easy-nLC 1000; Thermo Fisher, Waltham, MA, USA) and introduced into a Q Exactive mass spectrometer (Thermo Fisher). Finally, peptides were analyzed by MASCOT (www.matrixscience.com).

### Proteome analyses

Data analyses were undertaken using Proteome Discoverer 1.4 (Thermo Scientific) which incorporates the MASCOT search engine. The *Homo sapiens* database from Uniprot was downloaded on 15 August 2019 and human protein sequences were searched. Carbamidomethyl was used as the fixed modification, with oxidation as the dynamical modification. The maximum number of missed cleavages considered was two. Immunoprecipitation samples were prepared in three independent experiments. Analyses involved only proteins that were detected by MS at least twice.

## Supplementary Information

Supplementary Information.

## Data Availability

The datasets used and/or analysed during the current study are available from the corresponding author on reasonable request.
