# Supplementary material for: Spatiotemporal 7q11.23 protein network analysis implicates the role of DNA repair pathway during human brain development
Source: Sci Rep. 2021 Apr 15;11:8246. doi: 10.1038/s41598-021-87632-x (PMC8050238; doi:10.1038/s41598-021-87632-x)
Supplement: Supplementary file 1 — Supplementary Information. [file 41598_2021_87632_MOESM1_ESM.docx]

**Spatiotemporal 7q11.23 protein network analysis implicates the role of DNA repair pathway during human brain development**

Liang Chen^1^, Weidi Wang^1, 2^, Wenxiang Cai^1^, Weichen Song^1^, Wei Qian^1^, Guan Ning Lin^1,2^**^*^**

^1^ Shanghai Mental Health Center, Shanghai Jiao Tong University School of Medicine, School of Biomedical Engineering, Shanghai Jiao Tong University, Shanghai 200030, China

^2^ Shanghai Key Laboratory of Psychotic Disorders, Shanghai 200030, China

* Corresponding author: [nickgnlin@sjtu.edu.cn](mailto:nickgnlin@sjtu.edu.cn)

**Supplementary**

**Figure S1. Significant enriched KEGG pathways of seventy-one partners exclusively from the P4R3 network.** The top eight significant enriched biological process GO results of seventy-one partners of 7q11.23 CNV exclusively from the P4R3 network are shown in the figure.

**
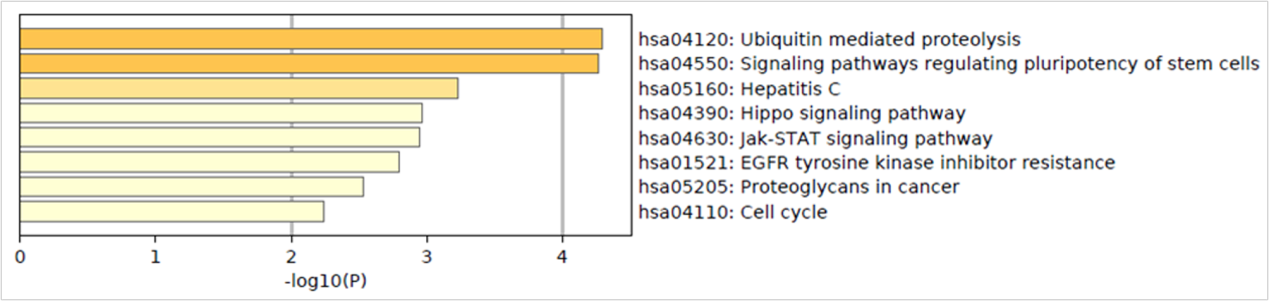
**

**Figure S2. Dynamic co-expression PPI networks of 7q11.23 CNV.** Dynamic networks of 7q11.23 CNV are shown in the Figure. 7q11.23 proteins are shown as red nodes, their co-expressed interacting partners as a gray node, and the PPIs between co-expressed genes at a particular developmental period are shown as gray edges. The nodes that lost all edges were removed from the corresponding networks.


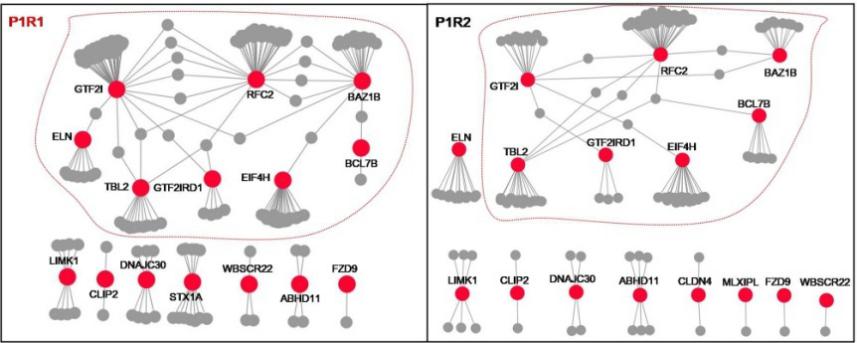


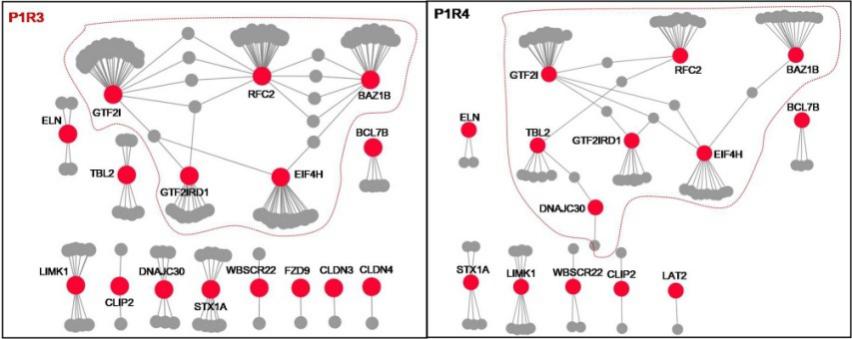


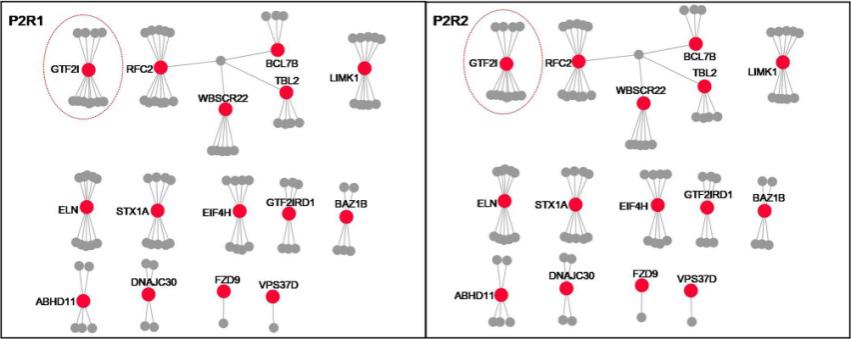


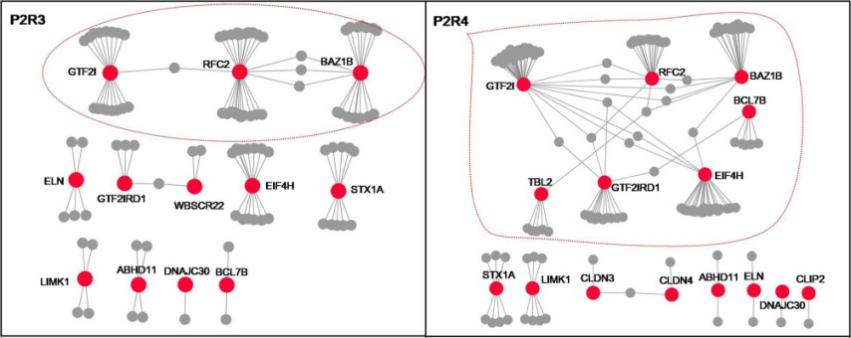


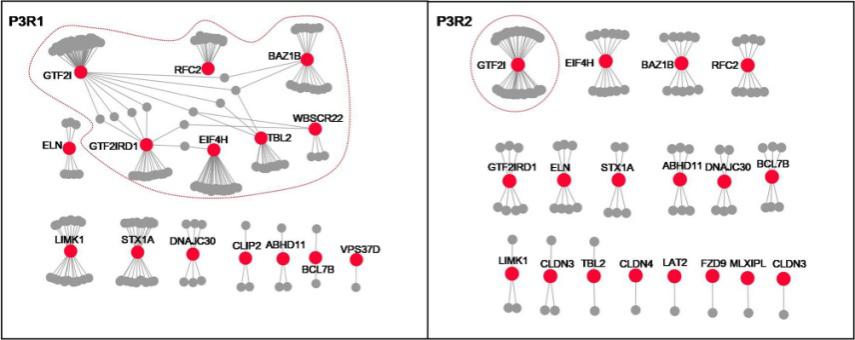


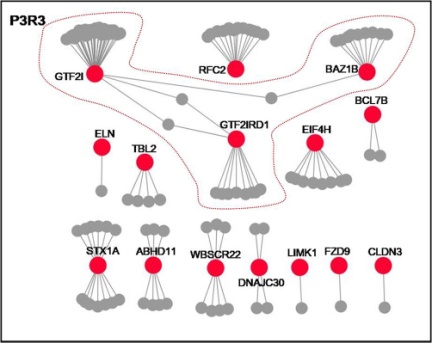


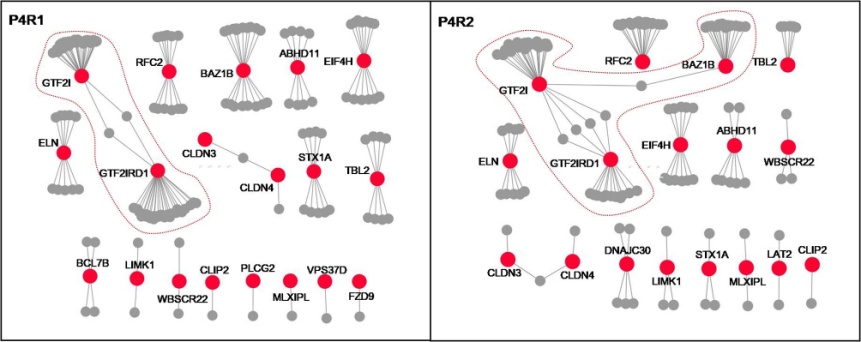


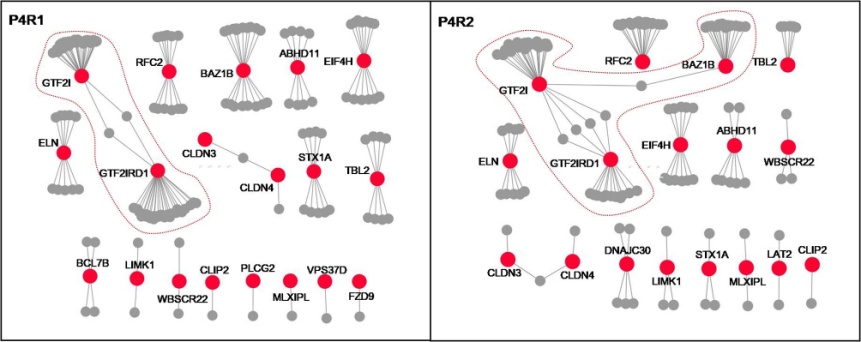


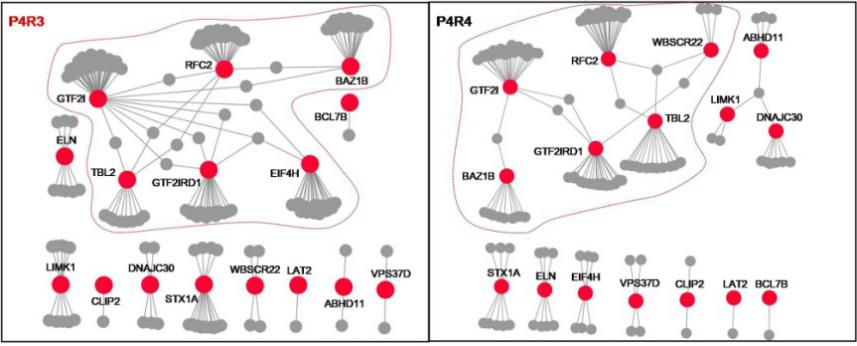


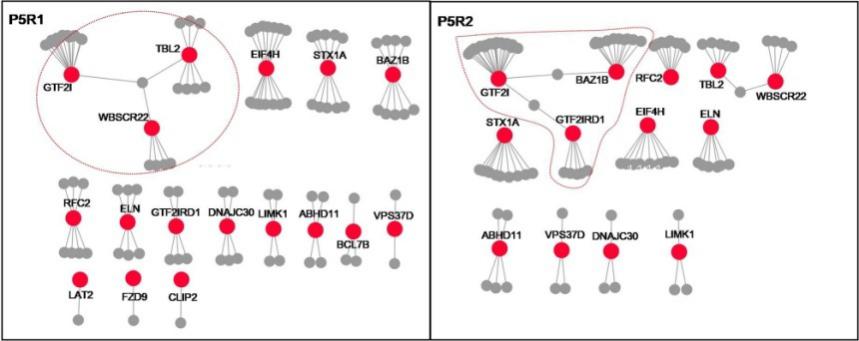


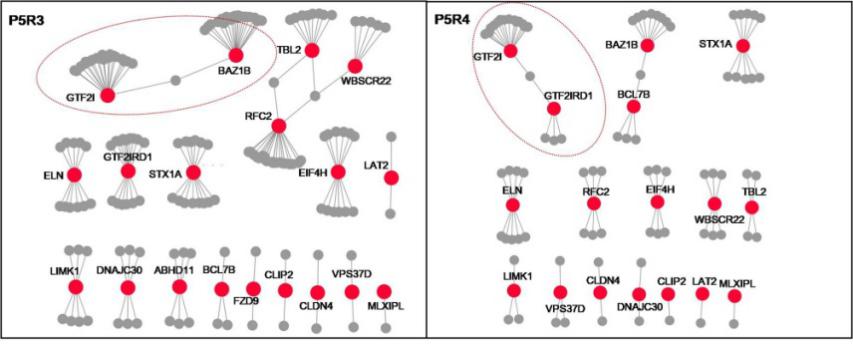


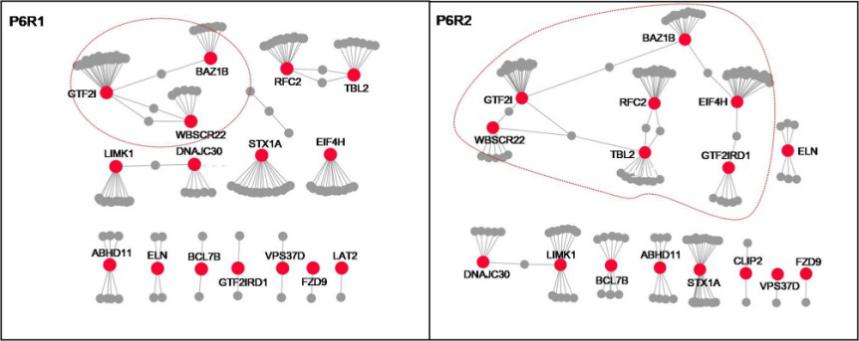


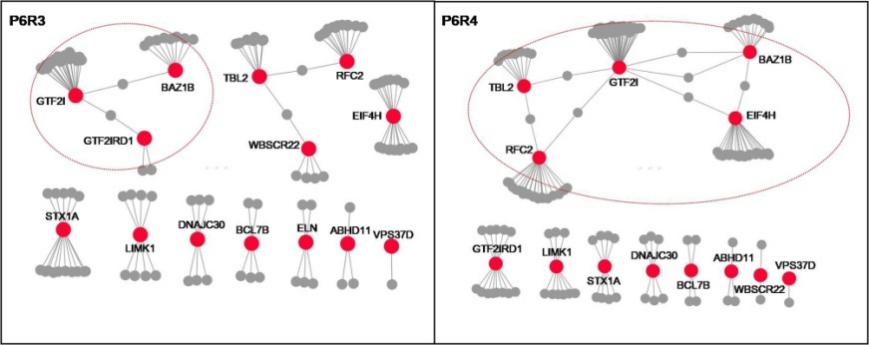


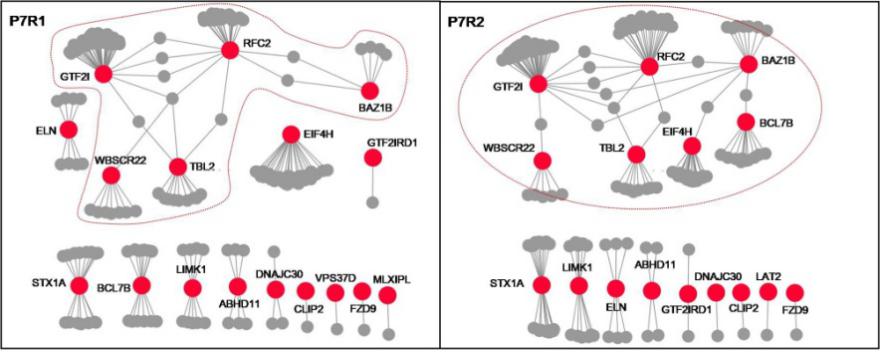


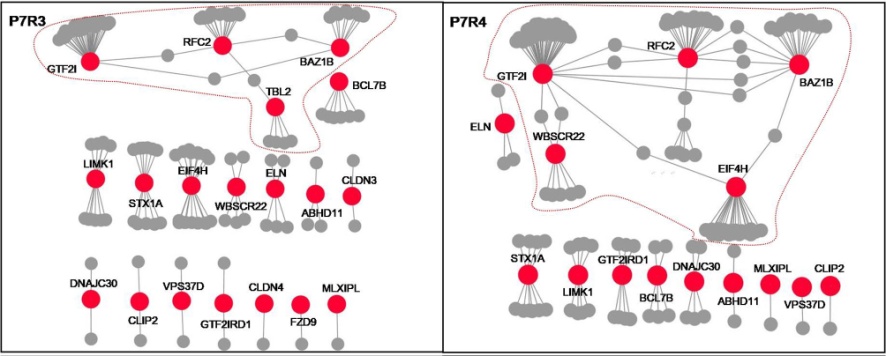


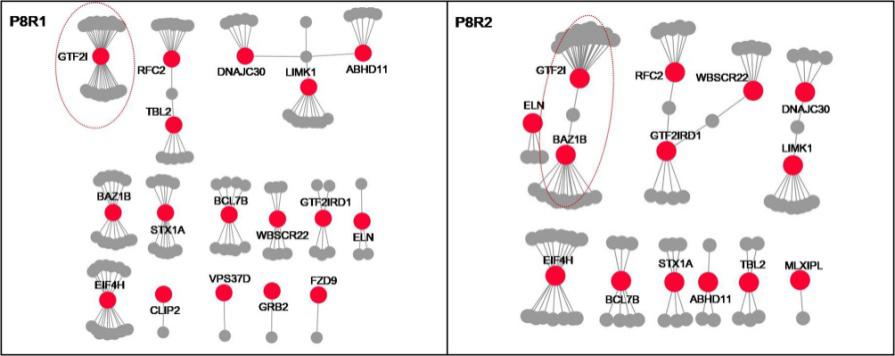


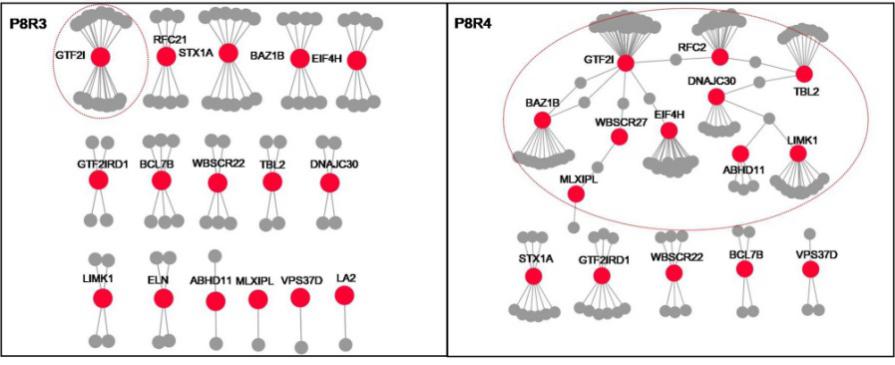


**Figure S3. Functional analyses of proteins within** **spatiotemporal co-expression PPI network of 7q11.23 CNV.** The top 20 significant enriched biological process GO results of proteins within dynamic networks are shown in the figure.
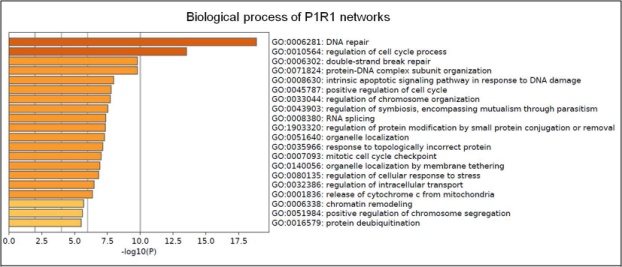

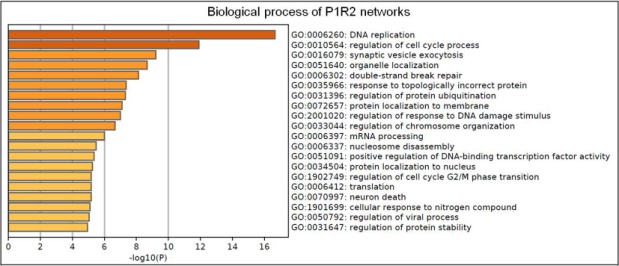


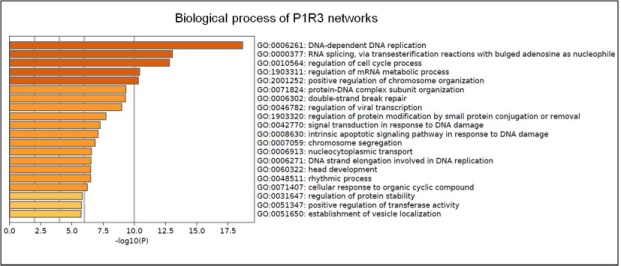

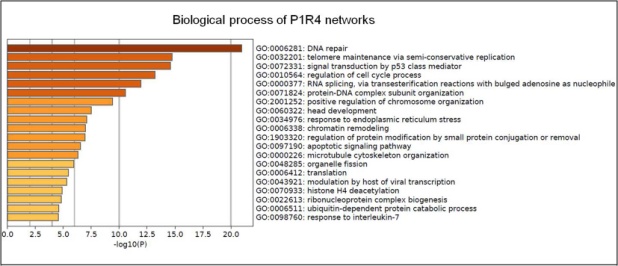


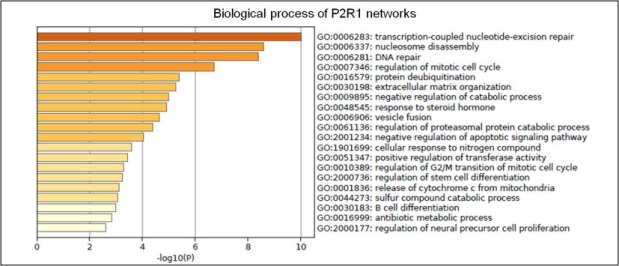

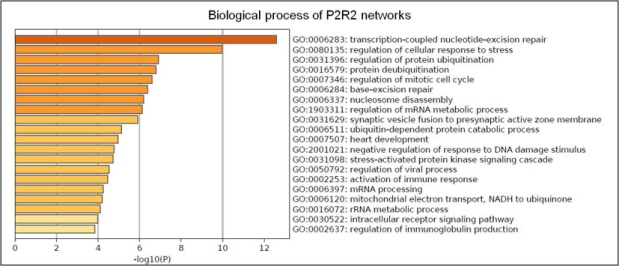


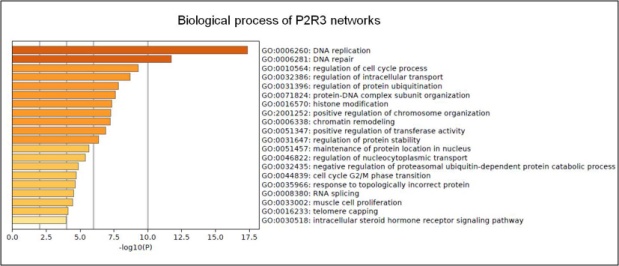

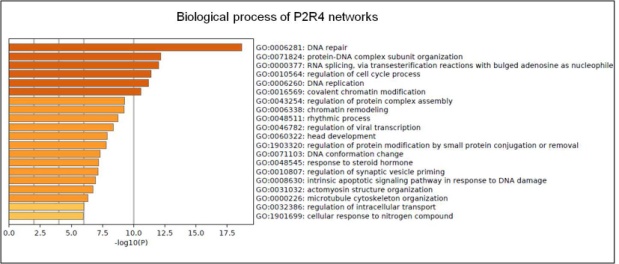


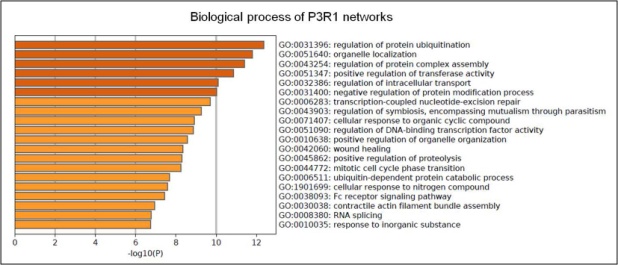

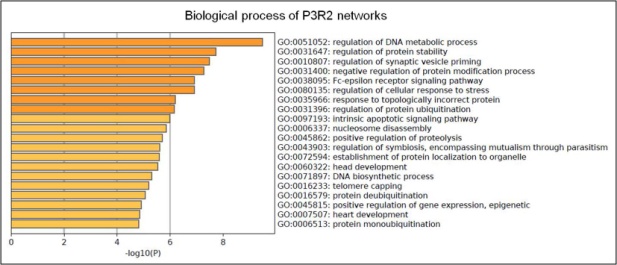


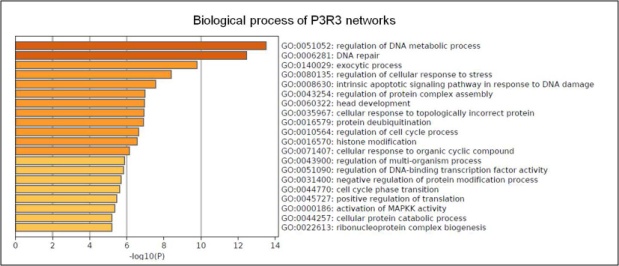


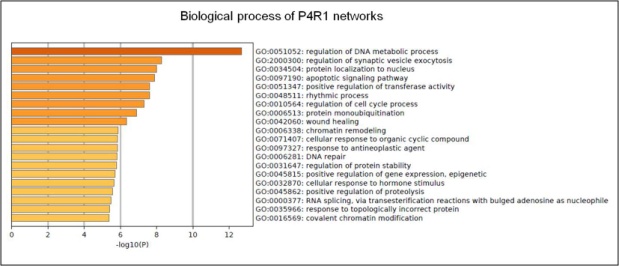

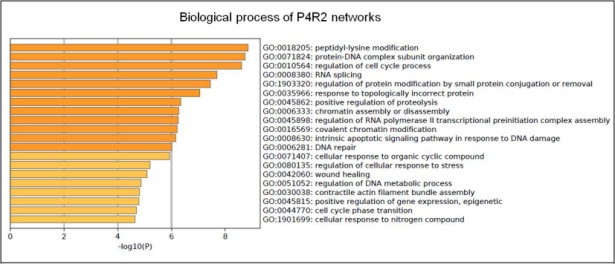


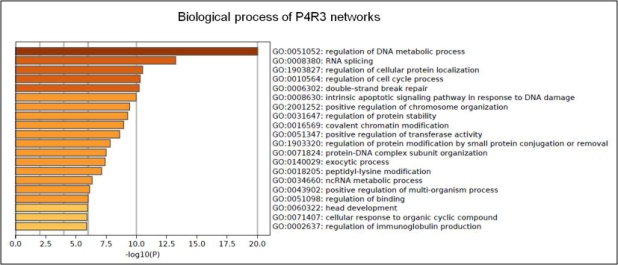

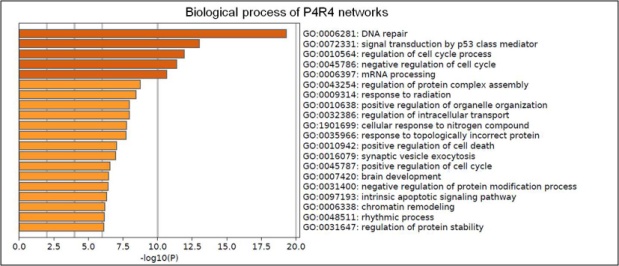


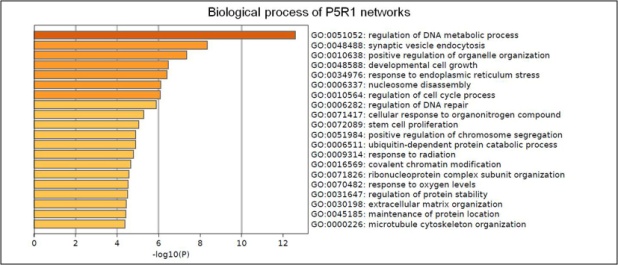

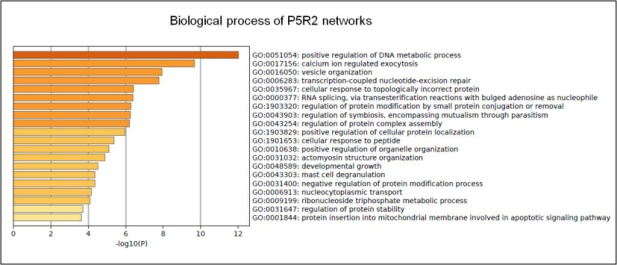


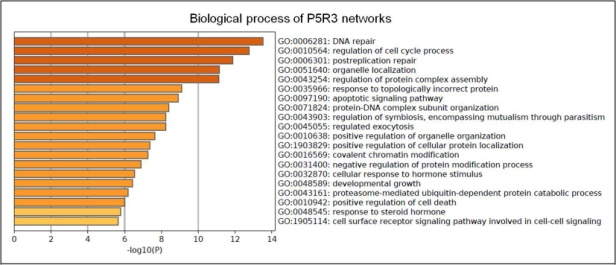

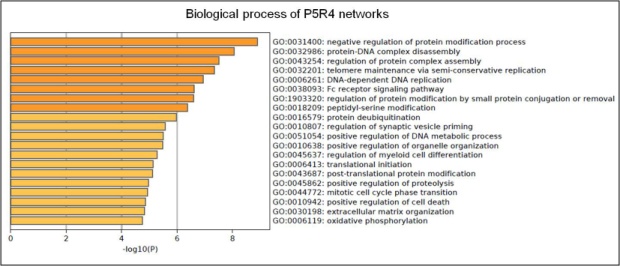


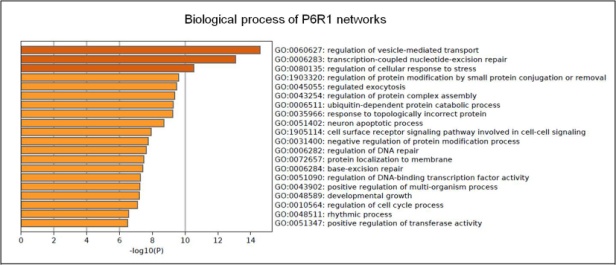

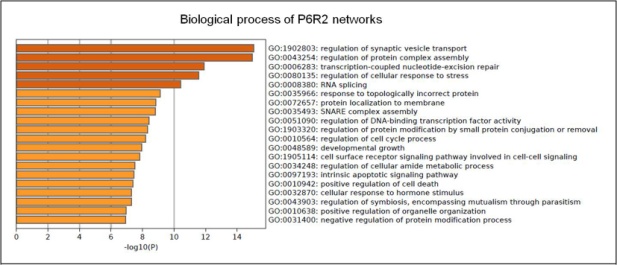


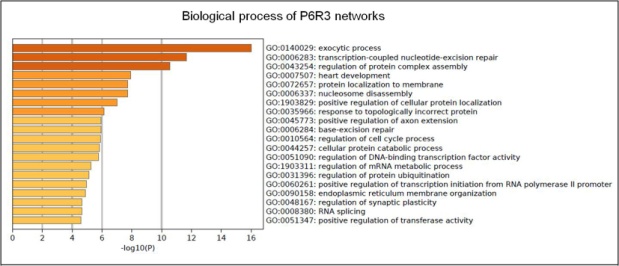

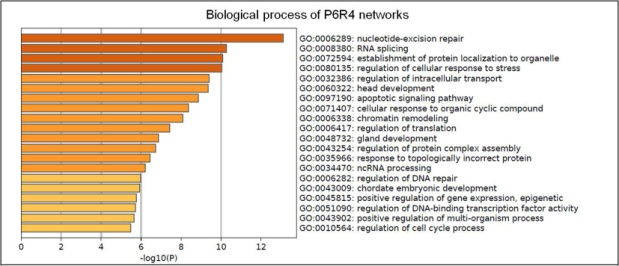

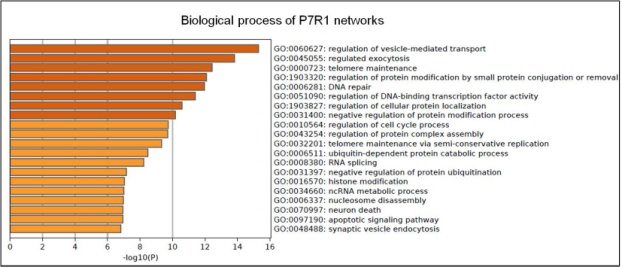

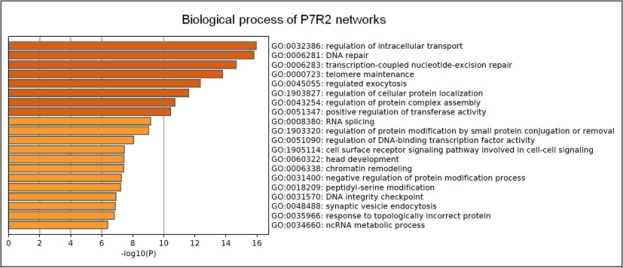


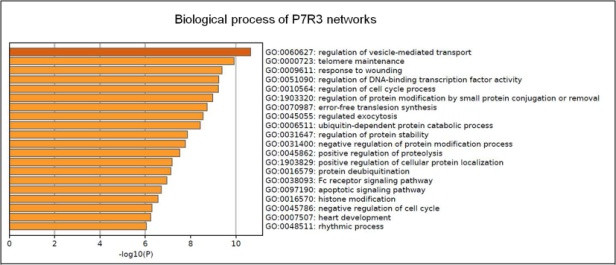

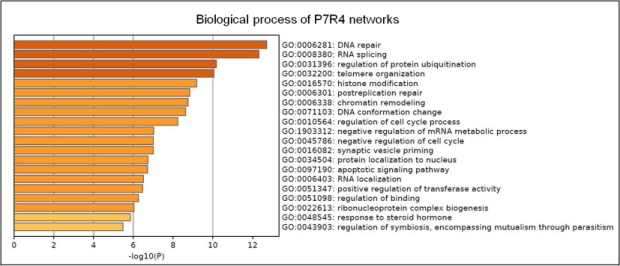


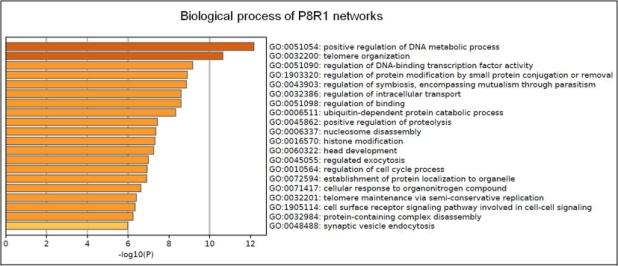

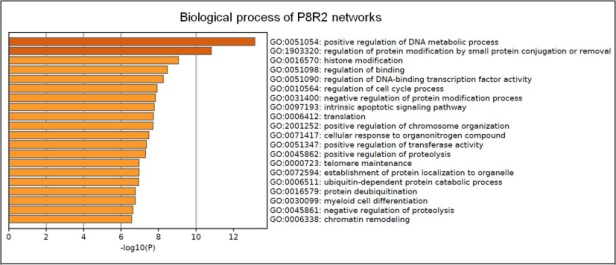


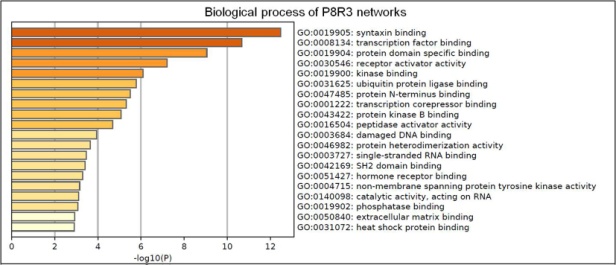

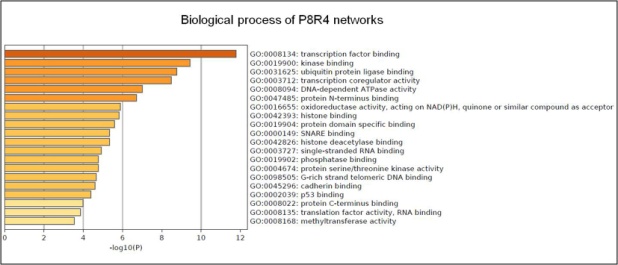


**Figure S4. SDS-PAGE separation of the immunoprecipitated proteins.**

**
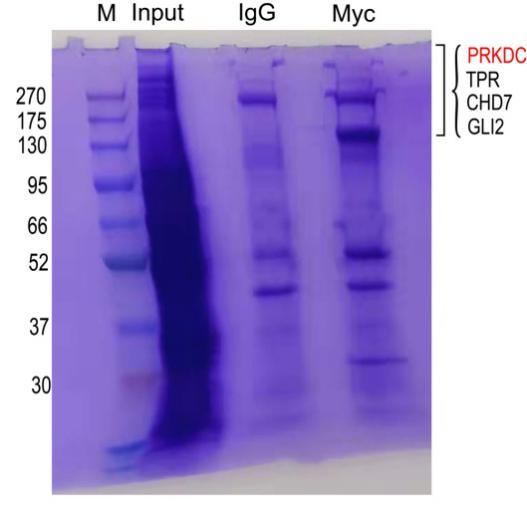
**

One-dimensional SDS-PAGE separation of the immunoprecipitated proteins using control IgG or Myc antibody. Several protein bands that are unique, which were identified by LC-MS/MS were listed on the right side of the panel.

**Table S1. Genes of 7q11.23 Copy number variation (CNV) region**

| **Gene ID** | **Gene symbol** | **Official full name** | **position (hg38)** | **size** |
| --- | --- | --- | --- | --- |
| 8468 | FKBP6 | FKBP prolyl isomerase 6 | chr7:73,328,161-73,358,625 | 30,465 |
| 8326 | FZD9 | frizzled class receptor 9 | chr7:73,433,778-73,436,120 | 2,343 |
| 9031 | BAZ1B | bromodomain adjacent to Zinc finger domain 1B | chr7:73,440,406-73,522,293 | 81,888 |
| 9275 | BCL7B | B-cell lymphoma 7 | chr7:73,536,356-73,557,690 | 21,335 |
| 26608 | TBL2 | transducin-beta like 2 | chr7:73,567,537-73,578,579 | 11,043 |
| 51085 | MLXIPL | MLX interacting protein like | chr7:73,593,194-73,624,540 | 31,347 |
| 155382 | VPS37D | VPS37D subunit of ESCRT-I | chr7:73,667,831-73,672,110 | 4,280 |
| 84277 | DNAJC30 | DnaJ heat shock protein family member C30 | chr7:73,680,918-73,683,453 | 2,536 |
| 114049 | WBSCR22 | Williams-Beuren syndrome chromosome region 22 | chr7:73,683,568-73,698,212 | 14,645 |
| 6804 | STX1A | syntaxin 1A | chr7:73,700,827-73,719,631 | 18,805 |
| 83451 | ABHD11 | abhydrolase domain containing 11 | chr7:73,736,094-73,738,867 | 2,774 |
| 1365 | CLDN3 | claudin 3 | chr7:73,768,997-73,770,270 | 1,274 |
| 1364 | CLDN4 | claudin 4 | chr7:73,827,744-73,832,684 | 4,941 |
| 155368 | WBSCR27 | methyltransferase like 27 | chr7:73,834,590-73,842,516 | 7,927 |
| 135886 | WBSCR28 | transmembrane protein 270 | chr7:73,861,159-73,865,890 | 4,732 |
| 2006 | ELN | elastin | chr7:74,027,789-74,069,907 | 42,119 |
| 3984 | LIMK1 | LIM domain kinase 1 | chr7:74,082,933-74,122,525 | 39,593 |
| 7458 | EIF4H | eukaryotic translation initiation factor 4H | chr7:74,174,245-74,197,101 | 22,857 |
| 7462 | LAT2 | linker for activation of T cells family member 2 | chr7:74,209,396-74,229,834 | 20,439 |
| 5982 | RFC2 | replication factor C, subunit 2 | chr7:74,231,499-74,254,389 | 22,891 |
| 7461 | CLIP2 | CAP-Gly domain containing linker protein 2 | chr7:74,289,475-74,405,943 | 116,469 |
| 9569 | GTF2IRD1 | GTF2I repeat domain containing 1 | chr7:74,453,970-74,602,604 | 148,635 |
| 2969 | GTF2I | general transcription factor II-I | chr7:74,650,231-74,760,692 | 110,462 |

**Table S2. Developmental brain period from the BrainSpan related to Figure 1**

| **stage** | **Description** | **Age** | **Developmental period** |
| --- | --- | --- | --- |
|  |  |  |  |
| 1 | Early fetal | 8-9 PCW | P1 |
| 2 | Early fetal | 10-12 PCW |  |
| 3 | Early mid-fetal | 13-15 PCW | P2 |
| 4 | Early mid-fetal | 16-18 PCW |  |
| 5 | Late mid-fetal | 19-23 PCW | P3 |
| 6 | Late fetal | 24-37 PCW | P4 |
| 7 | Early infancy | 0-5 M | P5 |
| 8 | Late infancy | 6-11 M |  |
| 9 | Early childhood | 1-5 Yr | P6 |
| 10 | late childhood | 6-11 Yr |  |
| 11 | Adolescence | 12-19 Yr | P7 |
| 12 | Young adulthood | 20-40 Yr | P8 |
|  |  |  |  |

**Table S3. Brain regions and the anatomical structures**

| **Brain Structure** | **Brain Region** |
| --- | --- |
| Posteroinferior (ventral) parietal cortex (IPC) | R1 |
| Primary auditory cortex (A1C) | R1 |
| Posterior (caudal) superior temporal cortex (STC) | R1 |
| Inferolateral temporal cortex (ITC) | R1 |
| Primary visual cortex (V1C) | R1 |
| Dorsolateral prefrontal cortex (DFC) | R2 |
| Ventrolateral prefrontal cortex (VFC) | R2 |
| Anterior (rostral) cingulate (medial prefrontal) cortex (MFC) | R2 |
| Orbital frontal cortex (OFC) | R2 |
| Primary motor cortex (M1C) | R2 |
| Primary somatosensory cortex (S1C) | R2 |
| Hippocampus | R3 |
| Amygdaloid complex | R3 |
| Striatum | R3 |
| The mediodorsal nucleus of the thalamus (MD) | R4 |
| Cerebellar cortex (CBC) | R4 |

**Table S4. Results of ANOVA test for interaction patterns of proteins from P1R3 and P4R3 networks.**

Interaction patterns of 7q11.23 CNV proteins across networks from different development periods. (P1:early fetal; P4: early mid-fetal) of the same brain region (R3: hippocampus, amygdala, and striatum) and the results of the ANOVA test related to Fig 4. Bold font indicates the p-value from the ANOVA test (p-value < 0.05)

| **CNV genes** | | **Interacting partners in P1R3 and P4R3** | | | | |
| --- | --- | --- | --- | --- | --- | --- |
| **Entrez** | **Official** | **Total**  **partners** | **unique to 1** | **network** | **shared by 2** | **networks** |
| **gene ID** | **symbol** |  | **count** | **Freq** | **count** | **Freq** |
| 8326 | FZD9 | 1 | 1 | 1.0000 | 0 | 0.0000 |
| 9031 | BAZ1B | 27 | 14 | 0.5185 | 13 | 0.4815 |
| 9275 | BCL7B | 5 | 5 | 1.0000 | 0 | 0.0000 |
| 26608 | TBL2 | 16 | 14 | 0.8750 | 2 | 0.1250 |
| 155382 | VPS37D | 2 | 2 | 1.0000 | 0 | 0.0000 |
| 84277 | DNAJC30 | 8 | 5 | 0.6250 | 3 | 0.3750 |
| 114049 | WBSCR22 | 5 | 4 | 0.8000 | 1 | 0.2000 |
| 6804 | STX1A | 19 | 13 | 0.6842 | 6 | 0.3158 |
| 83451 | ABHD11 | 2 | 2 | 1.0000 | 0 | 0.0000 |
| 1365 | CLDN3 | 1 | 1 | 1.0000 | 0 | 0.0000 |
| 1364 | CLDN4 | 1 | 1 | 1.0000 | 0 | 0.0000 |
| 2006 | ELN | 9 | 6 | 0.6667 | 3 | 0.3333 |
| 3984 | LIMK1 | 18 | 15 | 0.8333 | 3 | 0.1667 |
| 7458 | EIF4H | 34 | 21 | 0.6176 | 13 | 0.3824 |
| 7462 | LAT2 | 1 | 1 | 1.0000 | 0 | 0.0000 |
| 5982 | RFC2 | 45 | 30 | 0.6667 | 15 | 0.3333 |
| 7461 | CLIP2 | 3 | 3 | 1.0000 | 0 | 0.0000 |
| 9569 | GTF2IRD1 | 22 | 13 | 0.5909 | 9 | 0.4091 |
| 2969 | GTF2I | 63 | 34 | 0.5397 | 29 | 0.4603 |

| ANOVA Test |  |  |  |  |
| --- | --- | --- | --- | --- |
| Summary |  |  |  |  |
| Groups |  |  |  |  |
| Frequency of interactors | Count | Sum | Average | Variance |
| Unique to 1 network | 19 | 15.418 | 0.811 | 0.0349 |
| Shared by 2 network | 19 | 3.582 | 0.189 | 0.0349 |

| Result |  |  |  |  |  |
| --- | --- | --- | --- | --- | --- |
| Source of variation | SS | df | MS | F | **p-value** |
| Between Groups | 3.686 | 1 | 3.686 | 105.7 | **2.95 ×10^-12^***** |
| Within Groups | 1.256 | 36 | 0.035 |  |  |
|  |  |  |  |  |  |
| Total | 4.942 | 37 |  |  |  |

**Table S5. Results of ANOVA test for interaction patterns of proteins from P1R1 and P1R3 networks.**

Interaction patterns of 16p11.2 CNV proteins across networks from different brain regions (R1: parietal, temporal, and occipital cortex; R3:hippocampus, amygdala, and striatum) of the same developmental period (P1:early fetal;) and the results of the ANOVA test related to Fig 4. Bold font indicates the p-value from the ANOVA test (Not significant).

| **CNV genes** | | **Interacting partners in P1R1 and P1R3** | | | | |
| --- | --- | --- | --- | --- | --- | --- |
| **Entrez** | **Official** | **Total**  **partners** | **unique to 1** | **network** | **shared by 2** | **networks** |
| **gene ID** | **symbol** |  | **count** | **Freq** | **count** | **Freq** |
| 8326 | FZD9 | 1 | 0 | 1.000 | 1 | 4.000 |
| 9031 | BAZ1B | 22 | 3 | 0.136 | 19 | 0.864 |
| 9275 | BCL7B | 5 | 4 | 0.800 | 1 | 0.200 |
| 26608 | TBL2 | 16 | 12 | 0.750 | 4 | 0.250 |
| 84277 | DNAJC30 | 8 | 3 | 0.375 | 5 | 0.625 |
| 114049 | WBSCR22 | 3 | 1 | 0.333 | 2 | 0.667 |
| 6804 | STX1A | 16 | 10 | 0.625 | 6 | 0.375 |
| 83451 | ABHD11 | 4 | 4 | 1.000 | 0 | 0.000 |
| 1365 | CLDN3 | 1 | 1 | 1.000 | 0 | 0.000 |
| 1364 | CLDN4 | 1 | 1 | 1.000 | 0 | 0.000 |
| 2006 | ELN | 10 | 8 | 0.800 | 2 | 0.200 |
| 3984 | LIMK1 | 16 | 13 | 0.813 | 3 | 0.188 |
| 7458 | EIF4H | 29 | 10 | 0.345 | 19 | 0.655 |
| 5982 | RFC2 | 42 | 12 | 0.286 | 30 | 0.714 |
| 7461 | CLIP2 | 2 | 0 | 0.000 | 2 | 1.000 |
| 9569 | GTF2IRD1 | 15 | 13 | 0.867 | 2 | 0.133 |
| 2969 | GTF2I | 50 | 17 | 0.340 | 33 | 0.660 |

| ANOVA Test |  |  |  |  |
| --- | --- | --- | --- | --- |
| Summary |  |  |  |  |
| Groups | Count | Sum | Average | Variance |
| Frequency of interactors |  |  |  |  |
| Unique to 1 network | 17 | 10.469 | 0.616 | 0.122 |
| Shared by 2 network | 17 | 10.531 | 0.619 | 0.122 |

| Result |  |  |  |  |  |
| --- | --- | --- | --- | --- | --- |
| Source of variation | SS | df | MS | F | **p-value** |
| Between Groups | 0.111 | 1 | 0.1106 | 0.904 | 0.349 |
| Within Groups | 3.912 | 32 | 0.1223 |  |  |
|  |  |  |  |  |  |
| Total | 4.023 | 33 |  |  |  |

**Table S6. Parameters of spatiotemporal networks**

**(Number of CNV proteins≥5**).

**(1). Parameters of P1R1 network**

| **Entrez gene ID** | **Gene symbol** | **Radiality** | **Degree** | **Eccentricity** |
| --- | --- | --- | --- | --- |
| 2969 | GTF2I | 0.7951 | 48 | 5 |
| 9031 | BAZ1B | 0.7509 | 21 | 5 |
| 9275 | BCL7B | 0.5077 | 2 | 7 |
| 26608 | TBL2 | 0.6505 | 13 | 7 |
| 2006 | ELN | 0.5723 | 8 | 7 |
| 7458 | EIF4H | 0.5791 | 23 | 7 |
| 5982 | RFC2 | 0.7645 | 38 | 5 |
| 9569 | GTF2IRD1 | 0.6386 | 6 | 7 |

**(2). Parameters of P1R2 network**

| **Entrez gene ID** | **Gene symbol** | **Radiality** | **Degree** | **Eccentricity** |
| --- | --- | --- | --- | --- |
| 2969 | GTF2I | 0.743 | 15 | 5 |
| 5982 | RFC2 | 0.7824 | 36 | 5 |
| 9031 | BAZ1B | 0.6782 | 12 | 5 |
| 7458 | EIF4H | 0.5902 | 21 | 7 |
| 9569 | GTF2IRD1 | 0.5115 | 4 | 7 |
| 9275 | BCL7B | 0.5972 | 7 | 7 |
| 26608 | TBL2 | 0.6180 | 16 | 7 |

**(3). Parameters of P1R3 network**

| **Entrez gene ID** | **Gene symbol** | **Radiality** | **Degree** | **Eccentricity** |
| --- | --- | --- | --- | --- |
| 2969 | GTF2I | 0.7265 | 35 | 5 |
| 9031 | BAZ1B | 0.6125 | 20 | 5 |
| 7458 | EIF4H | 0.6667 | 25 | 5 |
| 5982 | RFC2 | 0.7009 | 34 | 5 |
| 9569 | GTF2IRD1 | 0.6581 | 11 | 5 |

**(4). Parameters of P1R4 network**

| **Entrez gene ID** | **Gene symbol** | **Radiality** | **Degree** | **Eccentricity** |
| --- | --- | --- | --- | --- |
| 2969 | GTF2I | 0.8444 | 29 | 7 |
| 5982 | RFC2 | 0.774 | 17 | 7 |
| 9031 | BAZ1B | 0.6823 | 15 | 11 |
| 7458 | EIF4H | 0.7944 | 13 | 9 |
| 9569 | GTF2IRD1 | 0.75 | 9 | 9 |
| 26608 | TBL2 | 0.6407 | 7 | 9 |

**(5). Parameters of P2R4 network**

| Entrez gene ID | Gene symbol | Radiality | Degree | Eccentricity |
| --- | --- | --- | --- | --- |
| 2969 | GTF2I | 0.8158 | 43 | 5 |
| 5982 | RFC2 | 0.7364 | 20 | 5 |
| 9031 | BAZ1B | 0.75 | 22 | 7 |
| 7458 | EIF4H | 0.7538 | 27 | 5 |
| 9569 | GTF2IRD1 | 0.7383 | 15 | 5 |
| 9275 | BCL7B | 0.5116 | 6 | 7 |
| 26608 | TBL2 | 0.5968 | 6 | 7 |

**(6). Parameters of P3R1 network**

| Entrez gene ID | Gene symbol | Radiality | Degree | Eccentricity |
| --- | --- | --- | --- | --- |
| 2969 | GTF2I | 0.8013 | 53 | 5 |
| 9031 | BAZ1B | 0.6424 | 18 | 7 |
| 7458 | EIF4H | 0.5939 | 29 | 7 |
| 9569 | GTF2IRD1 | 0.7315 | 17 | 5 |
| 26608 | TBL2 | 0.6443 | 14 | 7 |
| 114049 | WBSCR22 | 0.6753 | 5 | 5 |

**(7). Parameters of P4R3 network**

| Entrez gene ID | Gene symbol | Radiality | Degree | Eccentricity |
| --- | --- | --- | --- | --- |
| 2969 | GTF2I | 0.8007 | 57 | 3 |
| 9031 | BAZ1B | 0.6115 | 20 | 5 |
| 26608 | TBL2 | 0.5687 | 11 | 5 |
| 7458 | EIF4H | 0.6025 | 22 | 5 |
| 5982 | RFC2 | 0.6453 | 26 | 5 |
| 9569 | GTF2IRD1 | 0.598 | 20 | 5 |

**(8). Parameters of P4R4 network**

| **Entrez gene ID** | **Gene symbol** | **Radiality** | **Degree** | **Eccentricity** |
| --- | --- | --- | --- | --- |
| 2969 | GTF2I | 0.6801 | 24 | 7 |
| 5982 | RFC2 | 0.6643 | 27 | 9 |
| 114049 | WBSCR22 | 0.7297 | 5 | 7 |
| 26608 | TBL2 | 0.6405 | 15 | 9 |
| 9031 | BAZ1B | 0.5198 | 10 | 9 |

**(9). Parameters of P6R2 network**

| **Entrez gene ID** | **Gene symbol** | **Radiality** | **Degree** | **Eccentricity** |
| --- | --- | --- | --- | --- |
| 2969 | GTF2I | 0.7961 | 25 | 7 |
| 9031 | BAZ1B | 0.7686 | 14 | 7 |
| 26608 | TBL2 | 0.733 | 13 | 9 |
| 5982 | RFC2 | 0.6197 | 17 | 11 |
| 114049 | WBSCR22 | 0.6974 | 7 | 9 |
| 9569 | GTF2IRD1 | 0.5485 | 6 | 11 |
| 7458 | EIF4H | 0.6957 | 23 | 9 |

**(10). Parameters of P6R4 network**

| **Entrez gene ID** | **Gene symbol** | **Radiality** | **Degree** | **Eccentricity** |
| --- | --- | --- | --- | --- |
| 2969 | GTF2I | 0.7975 | 40 | 3 |
| 9031 | BAZ1B | 0.6106 | 13 | 5 |
| 26608 | TBL2 | 0.6012 | 11 | 5 |
| 5982 | RFC2 | 0.6386 | 23 | 5 |
| 7458 | EIF4H | 0.6417 | 23 | 5 |

**(11). Parameters of P7R1 network**

| **Entrez gene ID** | **Gene symbol** | **Radiality** | **Degree** | **Eccentricity** |
| --- | --- | --- | --- | --- |
| 2969 | GTF2I | 0.8333 | 49 | 5 |
| 9031 | BAZ1B | 0.4637 | 6 | 5 |
| 26608 | TBL2 | 0.6992 | 12 | 5 |
| 5982 | RFC2 | 0.7572 | 23 | 3 |
| 114049 | WBSCR22 | 0.6847 | 8 | 5 |

**(12). Parameters of P7R2 network**

| **Entrez gene ID** | **Gene symbol** | **Radiality** | **Degree** | **Eccentricity** |
| --- | --- | --- | --- | --- |
| 2969 | GTF2I | 0.7786 | 47 | 5 |
| 9031 | BAZ1B | 0.75 | 12 | 5 |
| 26608 | TBL2 | 0.6106 | 10 | 7 |
| 5982 | RFC2 | 0.729 | 27 | 5 |
| 114049 | WBSCR22 | 0.5515 | 6 | 7 |
| 7458 | EIF4H | 0.5877 | 23 | 7 |

**(13). Parameters of P7R4 network**

| **Entrez gene ID** | **Gene symbol** | **Radiality** | **Degree** | **Eccentricity** |
| --- | --- | --- | --- | --- |
| 2969 | GTF2I | 0.8526 | 53 | 5 |
| 9031 | BAZ1B | 0.7733 | 20 | 5 |
| 5982 | RFC2 | 0.7428 | 22 | 5 |
| 7458 | EIF4H | 0.7327 | 23 | 7 |

**(14). Parameters of P8R4 network**

| **Entrez gene ID** | **Gene symbol** | **Radiality** | **Degree** | **Eccentricity** |
| --- | --- | --- | --- | --- |
| 5982 | RFC2 | 0.7926 | 18 | 7 |
| 83451 | ABHD11 | 0.5243 | 4 | 13 |
| 3984 | LIMK1 | 0.5336 | 12 | 13 |
| 84277 | DNAJC30 | 0.644 | 8 | 11 |
| 155368 | WBSCR27 | 0.6788 | 2 | 11 |
| 2969 | GTF2I | 0.8124 | 34 | 9 |
| 7458 | EIF4H | 0.716 | 20 | 11 |
| 51085 | MLXIPL | 0.5406 | 2 | 13 |
| 26608 | TBL2 | 0.73112 | 11 | 9 |

**Table S7. Parameters of interacting partners from a functional module within P1R1, P1R3, and P4R3 networks.**

1. **Parameters of interacting partners from a functional module within P1R1**

| **Entrez gene ID** | **Gene symbol** | **Radiality** | **Degree** | **Eccentricity** |
| --- | --- | --- | --- | --- |
| 5591 | PRKDC | 0.8611 | 7 | 2 |
| 672 | BRCA1 | 0.7500 | 5 | 3 |
| 6117 | RPA1 | 0.7778 | 6 | 3 |
| 6118 | RPA2 | 0.7778 | 6 | 3 |
| 6119 | RPA3 | 0.7500 | 5 | 3 |
| 8452 | CUL3 | 0.6667 | 2 | 3 |
| 8841 | HDAC3 | 0.7778 | 4 | 2 |
| 7706 | TRIM25 | 0.7222 | 3 | 3 |
| 3191 | HNRNPL | 0.6389 | 2 | 3 |
| 9203 | ZMYM3 | 0.5278 | 2 | 3 |

1. **Parameters of interacting partners from a functional module within P1R3**

| **Entrez gene ID** | **Gene symbol** | **Radiality** | **Degree** | **Eccentricity** |
| --- | --- | --- | --- | --- |
| 5591 | PRKDC | 0.8864 | 7 | 3 |
| 672 | BRCA1 | 0.8636 | 5 | 2 |
| 6117 | RPA1 | 0.8864 | 7 | 3 |
| 8452 | CUL3 | 0.7500 | 3 | 4 |
| 3191 | HNRNPL | 0.8409 | 4 | 2 |
| 6119 | RPA3 | 0.7273 | 3 | 4 |
| 6118 | RPA2 | 0.8182 | 4 | 3 |
| 8841 | HDAC3 | 0.7045 | 2 | 4 |
| 9203 | ZMYM3 | 0.5227 | 1 | 4 |

1. **Parameters of interacting partners from a functional module within P4R3**

| **Entrez gene ID** | **Gene symbol** | **Radiality** | **Degree** | **Eccentricity** |
| --- | --- | --- | --- | --- |
| 6117 | RPA1 | 0.8750 | 6 | 2 |
| 8452 | CUL3 | 0.7708 | 3 | 3 |
| 672 | BRCA1 | 0.8125 | 4 | 3 |
| 7750 | ZMYM2 | 0.7292 | 3 | 4 |
| 9203 | ZMYM3 | 0.7500 | 4 | 4 |
| 3191 | HNRNPL | 0.7917 | 3 | 3 |
| 6118 | RPA2 | 0.5000 | 1 | 4 |
| 5591 | PRKDC | 0.8333 | 5 | 3 |
| 8841 | HDAC3 | 0.7292 | 3 | 4 |
| 6119 | RPA3 | 0.6458 | 1 | 3 |

**Table S8.** **Enrichment analysis of de novo mutations from 7q11.23 spatiotemporal networks**

| Number of genes within 7q11.23 | Number of | Number of autism risk genes | Fisher exact | **Benjamini-Hochberg** |
| --- | --- | --- | --- | --- |
| spatiotemporal networks | autism risk genes | in spatiotemporal networks | p-value | **adj p-value** |
| 635 | 239 | 20 | 0.00012 | **0.00072** |

| Number of genes within 7q11.23 | Number of | Number of developmental delays | Fisher exact | **Benjamini-Hochberg** |
| --- | --- | --- | --- | --- |
| spatiotemporal networks | developmental delay genes | genes in spatiotemporal networks | p-value | **adj p-value** |
| 635 | 1302 | 60 | 0.00302 | **p=0.00450** |

| Number of genes within 7q11.23 | Number of fragile X mental | Number of FMRP genes | Fisher exact | **Benjamini-Hochberg** |
| --- | --- | --- | --- | --- |
| spatiotemporal networks | retardation protein (FMRP) | in spatiotemporal networks | p-value | **adj p-value** |
| 635 | 839 | 45 | 0.00057 | **p= 0.00170** |

| Number of genes within 7q11.23 | Number of | Number of neurodegenerative disease genes | Fisher exact | **Benjamini-Hochberg** |
| --- | --- | --- | --- | --- |
| spatiotemporal networks | neurodegenerative disease genes | in spatiotemporal networks | p-value | **adj p-value** |
| 635 | 93 | 7 | 0.03124 | **0.03749** |
